# Temporal and spatial coordination of DNA segregation and cell division in an archaeon

**DOI:** 10.1073/pnas.2513939122

**Published:** 2025-10-15

**Authors:** Joe Parham, Valerio Sorichetti, Alice Cezanne, Sherman Foo, Yin-Wei Kuo, Baukje Hoogenberg, Arthur Radoux-Mergault, Eloise Mawdesley, Lydia Daniels Gatward, Jerome Boulanger, Ulrike Schulze, Anđela Šarić, Buzz Baum

**Affiliations:** ^a^Division of Cell Biology, Medical Research Council Laboratory of Molecular Biology, Cambridge CB2 0QH, United Kingdom; ^b^Institute of Science and Technology Austria, Klosterneuberg 3400, Austria

**Keywords:** cell division, checkpoint, archaea, evolution, chromosome segregation

## Abstract

Cells must coordinate DNA segregation with cytokinesis to ensure that each daughter cell inherits a complete genome. Here, we explore how DNA segregation and division are mechanistically coupled in archaeal relatives of eukaryotes, which lack Cyclin-dependent kinase (CDK)/Cyclins. Using live cell imaging, we first describe the series of sequential changes in DNA organization that accompany cell division in *Sulfolobus,* which computational modeling shows likely aid genome segregation. Through a perturbation analysis we identify a regulatory checkpoint which ensures that the compaction of the genome into two spatially segregated nucleoids only occurs once cells have assembled a division ring—which also defines the axis of DNA segregation. Finally, we show that DNA compaction and segregation depend, in part, on a ParA homologue, SegA, and its partner SegB, whose absence leads to bridging DNA. Taken together, these data show how regulatory checkpoints like those operating in eukaryotes aid high-fidelity division in an archaeon.

## Significance

A key event in any cell’s life is division, when one cell becomes two. Here, we explore how archaea coordinate DNA segregation with division. Our analysis identifies a regulatory decision point in the *Sulfolobus* cell cycle that functions like the eukaryotic spindle assembly checkpoint, in that it ensures that DNA segregation does not occur until cells have assembled the medial ring that defines the axis of DNA segregation. Furthermore, we identify a likely role for entropy in the disentanglement of the two nucleoids and a role for two proteins, SegA and SegB in DNA compaction and segregation. Taken together, these data reveal parallels and differences in the way cell division is regulated between archaea, like *Sulfolobus*, and eukaryotes.

The regulation of the eukaryotic cell division cycle is now relatively well understood in a variety of model systems, from yeast to humans (1). In eukaryotes, orderly cell cycle progression relies on waves of transcription (2), proteasome-mediated protein degradation (3), and oscillations in Cyclin-dependent kinase (CDK)-cyclin-dependent protein phosphorylation (4). These controls are aided by cell cycle checkpoints, which ensure that critical events in the cell cycle are completed before the initiation of subsequent steps (5). One of the best studied examples of this is the spindle assembly checkpoint, which ensures that spindle-dependent DNA separation and cytokinesis are not triggered until the system is in a state of readiness (6–8). In addition, to ensure that each nascent daughter cell inherits a complete copy of the parental cell’s genome, DNA segregation and cytokinesis must be spatially coordinated so that the division plane bisects the center of the spindle.

In different eukaryotes this coordination is achieved in distinct ways. In animal cells, for example, which undergo a relatively open mitosis, a signal emanating from overlapping microtubules at the center of the anaphase spindle triggers the assembly and contraction of an overlying actomyosin-based cytokinetic ring (9–11). Later, the midbody positions the ESCRT-III machinery to bring about abscission at a site adjacent to the center of the division bridge (12). Conversely, in budding yeast cells, which undergo a closed mitosis, in addition to the operation of a spindle checkpoint which ensures high fidelity DNA segregation (13), DNA segregation and cell division are coordinated by a checkpoint that monitors spindle position (14).

Outside of eukaryotes, there is little evidence to support the operation of similar cycle checkpoints. While it is often assumed that this reflects the lack of clear temporal separation between DNA replication, DNA segregation, and cell division in prokaryotes (15), including in some archaea (16), many of the closest archaeal relatives of eukaryotes, including *Sulfolobus acidocaldarius*, possess an orderly cell division cycle (17). This involves waves of transcription (18) and proteasome-dependent protein degradation (19). Furthermore, despite lacking close homologues of CDK-Cyclins (19), *Sulfolobus* cells use eukaryotic-like Cdc6/Orc1 proteins to fire multiple origins of DNA replication once per cell cycle (20, 21) and, like human cells, employ composite ESCRT-III polymers to trigger abscission at the end of each cell cycle (22–26). This suggests the possibility that there may be deeply conserved mechanisms and/or general principles of cell cycle control that have yet to be revealed.

At the same time, some of the machinery *Sulfolobus* cells use to control critical events in their cell cycle are profoundly different from those operating in eukaryotes. For example, *Sulfolobus* cells use an archaeal specific protein CdvA rather than ESCRT-I and II complexes to nucleate ESCRT-III polymer formation (27–30). In addition, while *Sulfolobus* cells possess a Rad50-like SMC protein, called Coalescin (31), they appear to lack functional homologues of the structural maintenance of chromosome (SMC) proteins Cohesin and Condensin that help drive DNA individualization in bacteria and eukaryotes (32, 33). In addition, they lack homologues of tubulin, while possessing a ParA-like protein SegA which, based on in vitro work, has been postulated to work together with a DNA binding-partner protein, SegB, to drive genome segregation (34, 35).

Since it is not known how archaea coordinate DNA segregation and cell division, in this paper we use *Sulfolobus* as an experimentally tractable model system to explore how these different processes function together to ensure that each daughter cell inherits a full copy of the genome at the end of each cell cycle (36). Through this work, we reveal a complex choreographed set of changes in genome organization that accompany division. In addition, we identify a regulatory decision point in the *Sulfolobus* cell cycle that, like the spindle assembly checkpoint in eukaryotes, acts to ensure that cells do not commit to cell division until everything is in place—implying the existence of common rules that aid cell division in different organisms across the tree of life.

## Results

### DNA Segregation and Cell Division Are Temporally Coordinated.

**Fig. 1.**
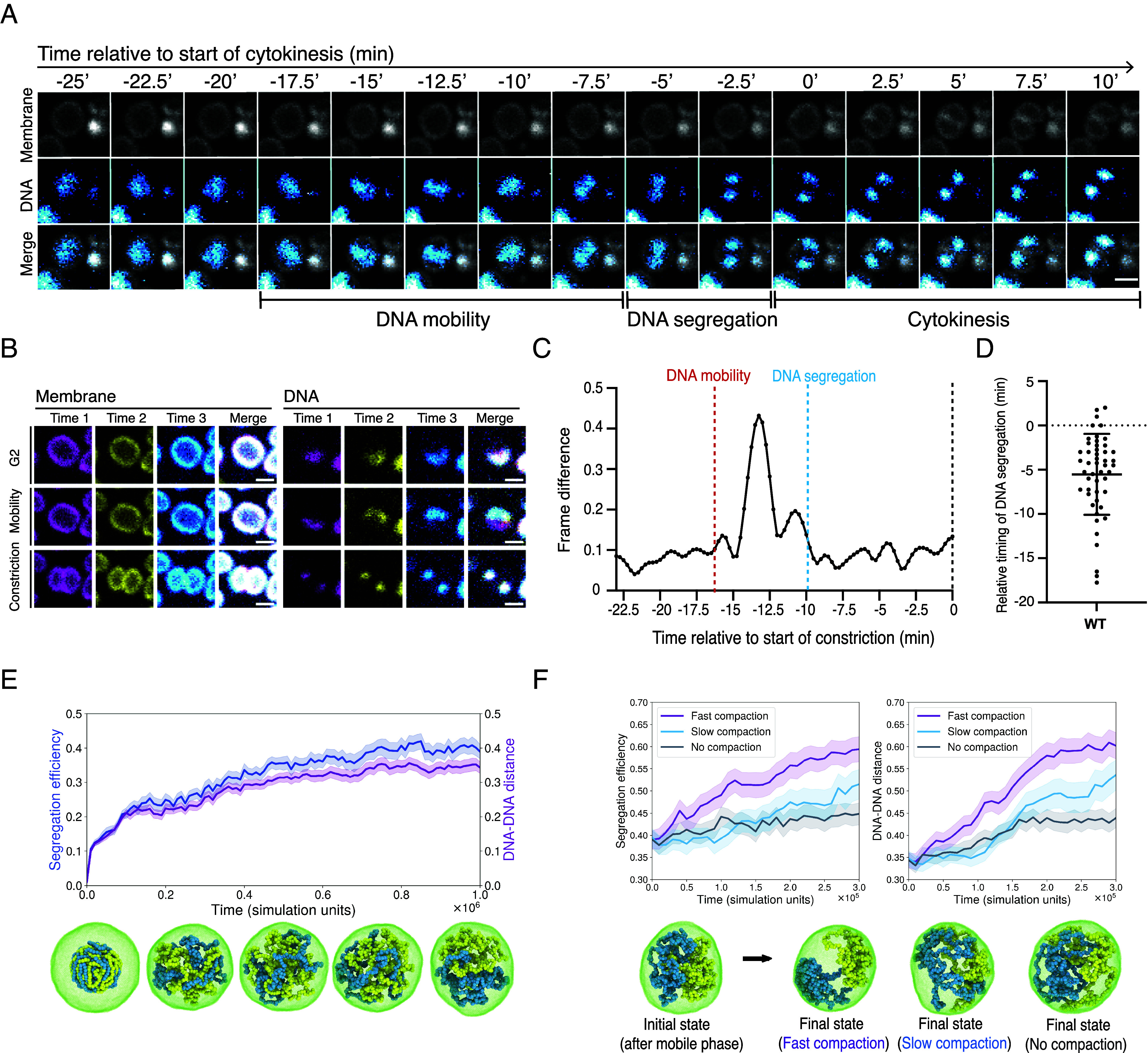
DNA segregation and cell division are temporally coordinated. (*A*) Montage of a dividing wildtype cell with membrane and DNA visualized from late G2 through DNA segregation and cell division. (Scale bar, 1 μm.) (*B*) Single slices and colored overlays of membrane and DNA taken from three frames (in magenta, yellow, and cyan) of a live imaging analysis of a wildtype cell during late G2 (prior to release from the membrane); prior to DNA segregation; and after the onset of cell constriction. (Scale bar, 1 μm.) (*C*) An analysis of DNA motion over time in a wildtype cell made by comparing the DNA signal across frames to highlight differences. Annotations have been added by hand to highlight the discrete phases of DNA mobility, DNA segregation, and the onset of cytokinesis as determined by eye. (*D*) Quantification of the timing of DNA segregation relative to timing of the first frame of cell constriction in cytokinesis (0) in wildtype cells (n = 49, N = 3 biological replicates). Mean plotted with error bars denoting ± SD. (*E*) Segregation efficiency and normalized distance between the centers of mass of the chromosomes for multiple simulations of cells during the mobile phase (n = 50; shaded area: SE) and example snapshots for one of the simulated systems (10% DNA volume fraction). The cell membrane is in green, while the two chromosomes are yellow and blue, respectively. The snapshots were taken at time intervals of 2.5 × 10^5^τ (simulation time units). (*F*) Segregation efficiency (*Left*) and normalized distance between the centers of mass of the chromosomes (*Right*) for simulations of cells undergoing fast and slow DNA compaction, compared with the no compaction control (n = 50; shaded area: SE). The data show simulations run using a 10% DNA volume fraction. The snapshots represent the common initial state (*Left*) and the final states (*Right*) of three simulations run with fast, slow, or no compaction.

To investigate the spatial and temporal coordination of DNA segregation and cell division in *Sulfolobus*, we began by using live imaging to follow wildtype cells (DSM 639) as they progressed through the cell cycle. Cell Mask Deep Red Plasma Membrane Stain and SYBR Safe were used to label the cell membrane and DNA respectively, and cells were imaged at 15 s intervals at 75 °C using our upgraded Sulfoscope set up (37, 38). This revealed a dynamic series of changes in DNA localization, compaction, and segregation that accompany the progression of cells from G2 into division (Fig. 1*A*).

For most of the cell cycle, the DNA appeared stably localized to a discrete site close to the cell membrane (Fig. 1*A* and *SI Appendix*, Fig. S1). However, as cells progress toward division, the DNA dissociated from the membrane—becoming diffuse and mobile in the process (Movies S1 and S2). After a variable length of time (average 18 ± 9 min) (*SI Appendix*, Fig. S2*A*), this “mobile phase” ended when the DNA compacted to form two spatially separate foci. This process of nucleoid individualization was rapid, often occurring within the 15 s interval separating consecutive frames of the movie (*SI Appendix*, Fig. S2 and Movies S1 and S2). Once compacted, the two nucleoids remained relatively stationary. Later, during cytokinesis, the nucleoids reassociated with the membrane. In the majority of cases the DNA bound the membrane at opposing poles of the dumbbell-shaped doublet. However, the timing at which the DNA moved onto the poles was variable. In some cells, DNA localized to cell poles immediately after segregation, whereas in other cells, we observed the DNA becoming associated with the membrane during cytokinesis.

To visualize how DNA organization changes as cells progress from G2 of the cell cycle, through division, and into G1 by other means, we colored the DNA and membrane signals from three near consecutive frames in magenta, yellow, and blue, before overlaying the images. By monitoring the intensity of the white signal, we were then able to visualize the extent to which the DNA moved in late G2 cells, following dissociation of DNA from the membrane, and following the onset of cytokinesis (Fig. 1*B*). The DNA appeared largely stationary in G2 cells (white), highly mobile in cells preparing for division (multicolored), before becoming rapidly compacted to form two, spatially separate, but stationary DNA foci (white). As an automated quantitative measure of DNA dynamics in cells progressing from G2 into division, we also used an image analysis pipeline (*Materials and Methods*) to calculate the difference in the DNA signal at each pixel over time in the subset of dividing cells that did not move during imaging (Fig. 1*C*). Consistent with the observations described above, a significant peak in DNA motion was visible prior to the onset of DNA compaction and individualization (Fig. 1*C*), with little movement thereafter.

Importantly, the live imaging data also revealed a striking temporal correlation between the compaction of DNA into two spatially segregated nucleoids and the onset of cell division. In almost all cases, DNA segregation was complete before cytokinesis—occurring an average of 5.5 min before the first visible cell constriction (Fig. 1*D*). This implies strict temporal coordination between the two processes. Furthermore, DNA compaction and cell division were also coordinated in space, since the axis defined by the two separated nucleoids taken immediately after DNA segregation was consistently oriented at ~90° (92 ± 10.3°) to the plane of the future cytokinetic furrow (*SI Appendix*, Fig. S2*B*). This was confirmed using immunofluorescence in which the angle of the segregated nucleoids was measured relative to the axis of the nonconstricted CdvB1 ring (*SI Appendix*, Fig. S2*C*). Finally, cytokinesis itself tended to be fast (~6.6 ± 2.5 min)–and was considerably less variable in duration than the mobile phase (Fig. 1*A* and *SI Appendix*, Fig. S2*A*). Taken together, these data reveal that cell division in *Sulfolobus* is accompanied by a complex choreographed series of changes in DNA organization. In addition, they reveal a strong correlation between the axis along which the DNA is separated and compacted, and the placement of the cytokinetic furrow—implying the strict coordination of DNA segregation and division in both space and time.

As a way of assessing the likely function of the changes in DNA organization observed via live cell imaging, we developed a computational model of the process in which two circular DNA molecules (modeled as bead-spring polymers) were confined inside a vesicle, bounded by a thin membrane (Fig. 1*E*). To begin, we used this model to analyze the role of the mobile phase in DNA segregation. Earlier theoretical studies of bacterial DNA segregation had suggested that the weak entropic repulsion between overlapping chromosomes would be sufficient to lead to their segregation (39–41). However, these works also highlighted the importance of an elongated cell shape for efficient entropic segregation, implying that little to no segregation would occur via this mechanism in spherical cells (41).

To take a fresh look at this problem and investigate whether entropic forces can lead to significant segregation in spherical cells, like those of *Sulfolobus,* we initiated simulations with DNA in a compact state (representing volume fractions of either 5, 10, or 20%). We then let the DNA relax and undergo Brownian motion, while allowing chromosome strands to cross with a small energy penalty, in order to model the function of Topoisomerase-II. To assess the success of nucleoid segregation in simulations, we employed two different metrics, both of which tended to give similar results. The first, which we term “segregation efficiency,” was obtained using Linear Discriminant Analysis. This quantity is equal to 0 for a perfectly mixed configuration and 1 for a perfectly segregated one. The second measure defines the distance between the centers of mass of the two chromosomes, normalized by the vesicle radius. By analyzing simulations in this way, we were able to show that entropic forces are sufficient to drive the partial segregation of chromosomes in a spherical vesicle in a manner that is improved by lowering the DNA volume fraction (41) (Fig. 1*E* and *SI Appendix*, Fig. S3). When acting alone, however, these entropic forces were not sufficient to induce full DNA segregation (Fig. 1*E*), since they are unable to maintain the fully individualized state.

To explore whether the rapid compaction of DNA observed by live imaging might facilitate DNA segregation under these conditions (Fig. 1), we modeled this process by introducing a weak nonspecific attraction between all polymer beads, which was inhibited in a plane at the boundary of the two nucleoids to ensure alignment of the division machinery with the axis of DNA compaction (see below). After a period of entropic separation sufficient to reach equilibrium, we then compared the impact of slow or fast compaction on DNA segregation, relative to a no compaction control in simulations in which the DNA was allowed to re-associate with the membrane at late stages. While fast compaction of the DNA was not sufficient to induce complete DNA segregation, it significantly improved the quantitative outcome of simulations (Fig. 1*F* and *SI Appendix*, Fig. S3) resulting in a segregation efficiency of ~60%. These data imply that the different phases of choreographed DNA movement (DNA mobility, rapid DNA compaction, and the reassociation of DNA with the membrane that brings the cycle to a close) likely contribute to DNA segregation in *Sulfolobus*, while also making it clear that other mechanisms not included in this simple coarse-grained model are likely required to ensure that DNA segregation is robust, and goes to completion.

### The ESCRT-III Ring Couples DNA Segregation to Cell Division.

Having explored how dynamic changes in DNA organisation likely contribute to DNA segregation, we next sought to determine how DNA segregation, division ring assembly, and cytokinesis are coordinated in the cell. This requires aligning the axis of DNA segregation with the plane of cytokinesis. We considered two alternative hypotheses by which this might occur: i) The machinery driving DNA segregation defines the site of the future division plane (as the spindle does in human cells), or ii) the cytokinetic machinery sets the division plane to ensure the coordination of DNA segregation and cytokinesis. To ascertain which of these hypotheses best reflects the picture in dividing *Sulfolobus* cells, it was necessary to determine the relative timing of different steps in the process of division ring assembly with respect to the observed changes in DNA organization using immunofluorescence.

**Fig. 2.**
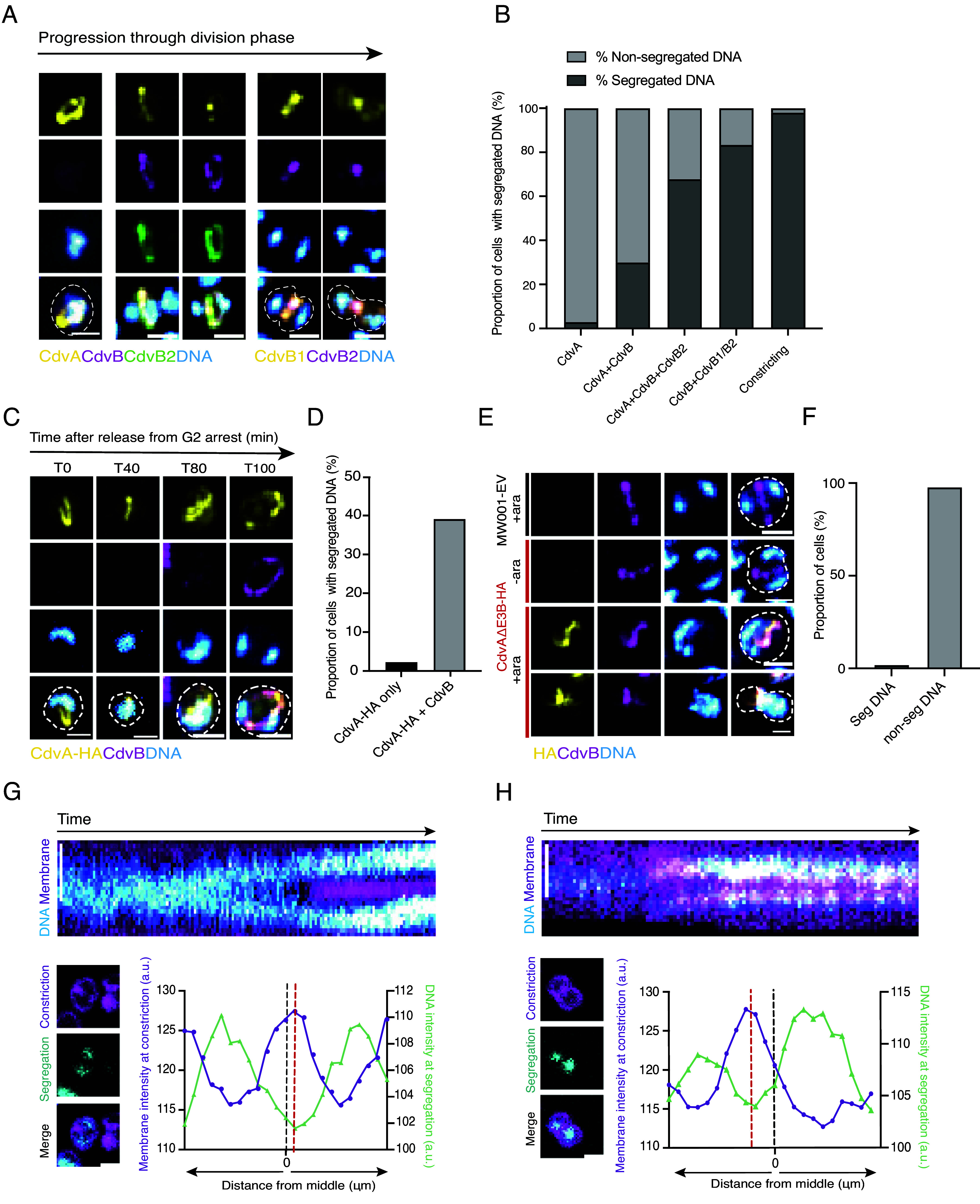
The ESCRT-III ring spatially and temporally couples DNA segregation to cell division. (*A*) Panel shows representative immunofluorescence images of wildtype DSM639 cells in different stages of cell division phase: cell with CdvA only ring; cell with CdvA+CdvB+CdvB2 ring; cellwith CdvB+CdvB2 ring; and cells in early and late constriction with just CdvB1 and CdvB2 rings. (*B*) Quantification of DNA segregation (proportion of cells with segregated vs. nonsegregated DNA) in wildtype cells with sequential ring compositions CdvA only (n = 63), CdvA+CdvB (n = 10), CdvA+CdvB+CdvB2 (n = 28), CdvB+CdvB1 or CdvB2 (n = 90), and constricting cells (n = 65) (from 3 biological replicates). (*C*) Representative immunofluorescence images of CdvA-HA positive cells at successive time points after early induced expression in a G2 arrest. (*D*) Quantification of DNA segregation following early induction of MW001-CdvA-HA in a G2 arrest (n = 66). (*E*) Representative immunofluorescence images of the MW001-EV control and cells overexpressing CdvA_ΔE3B_-HA before and after 4-h arabinose induction. (*F*) Quantification of DNA segregation in cells expressing CdvA_ΔE3B_-HA after 4-h arabinose induction (n = 344) (from 3 biological replicates). (*G* and *H*) Kymographs of two wildtype cells taken from movie (top). Images below show stills of the membrane and DNA from the start of constriction and from the first frame after DNA segregation, respectively, together with a merge of the two. Plots show membrane intensity at an early stage of constriction overlaid with DNA intensity from the first frame of DNA segregation, where 0 marks the geometric middle of the cell. All scale bars, 1 μm.

To begin this analysis, we extended our previous description of the steps in the pathway of ESCRT-III ring assembly (19, 24, 37) by determining the timing of CdvA expression and ring formation. Cells were fixed at 10 to 20-min intervals following release from a G2 arrest, stained for CdvA and CdvB, and analyzed by flow cytometry. By gating cells with a 2N DNA content, the levels of CdvA could be compared with those of CdvB as cells progressed toward division. By this measure, levels of CdvA were found to rise 10 to 20 min before levels of CdvB (*SI Appendix*, Fig. S4 *A* and *B*). Furthermore, using confocal microscopy, ring-like CdvA structures were seen forming at mid cell prior to formation of CdvB, B1, and B2 rings (Fig. 2*A* and *SI Appendix*, Fig. S4*C*). These data support the previously proposed idea that CdvA acts as a template for the recruitment of CdvB (27, 28).

Next, to investigate the timing of DNA segregation in cells relative to ESCRT-III ring recruitment, we fixed populations of *Sulfolobus* cells with ethanol and stained them with combinations of CdvA, CdvB, CdvB1, and CdvB2 antibodies to visualize the various stages of division. In cells with CdvA-only rings, this microscopy-based analysis revealed that the DNA formed a single diffuse mass, with only 3% of cells displaying two individualized DNA foci. In the rare cells with a composite CdvA/CdvB ring, the proportion of cells with two DNA foci rose significantly to 30%. This increased further to 68% with the recruitment of ESCRT-III homologs CdvB1 and CdvB2. In cells that possessed CdvB and CdvB1 or B2 rings, but which lacked a visible CdvA ring, the proportion of cells with two spatially separated DNA masses reached 83%. Finally, 98% of constricting wildtype cells were found to possess two individualized DNA foci (Fig. 2*B* and *SI Appendix*, Fig. S4*D*). These data reveal a tight temporal coupling of DNA segregation and cytokinesis. In addition, the data suggest that, in addition to its role as a template for the recruitment of the ESCRT-III division machinery, CdvA may also serve as a spatial marker that helps to define the plane of DNA separation.

Since DNA segregation follows the recruitment of CdvB to the CdvA ring, we considered several possible modes of regulation. First, the process might be triggered by some unknown target of the predivision wave of gene expression, with some delay relative to CdvA. Alternatively, the cue for DNA segregation might depend on assembly of the CdvA ring or on formation of the ESCRT-III division ring itself. To distinguish between these different possibilities, we asked whether the premature accumulation of CdvA protein was sufficient to induce DNA segregation. To do so, we overexpressed full-length CdvA tagged with HA from an inducible arabinose promoter in G2 arrested cells. Upon release from the arrest, cells were fixed and stained at 20-min intervals as they exited G2 and entered division. Strikingly, under these conditions, ectopically expressed CdvA was able to form linear, ring-like structures a full 80 min before the rise of endogenous CdvA expression in the MW001-empty vector control (Fig. 2*C* and *SI Appendix*, Fig. S4*E*). The presence of CdvA ring-like structures, however, was not sufficient to induce DNA segregation. Instead, significant DNA segregation was only observed 100 min postrelease, coincident with the recruitment of CdvB to the CdvA ring (Fig. 2*D*).

Next, to determine whether DNA segregation can occur in cells that are unable to complete ring assembly, we ectopically expressed a truncated version of CdvA tagged with HA (hereafter named CdvA_ΔE3B_) that lacks the CdvB-binding E3B domain (27, 28) (*SI Appendix*, Fig. S5*A*). This was sufficient to prevent the formation of complete CdvB rings, leading to the formation of abnormal linear CdvA positive structures that likely contain both endogenous CdvA as well as the CdvA_ΔE3B_ mutant protein. While these linear structures were able to recruit a portion of the cellular pool of CdvB, the expression of CdvA_ΔE3B_ almost completely blocked the recruitment of CdvB1 (*SI Appendix*, Fig. S5 *B* and *C*). As a consequence, cells expressing truncated CdvA were unable to divide, as evidenced by both the accumulation of large cells that had failed division (*SI Appendix*, Fig. S5*D*) and by the decrease in the percentage of 1N cells observed by flow cytometry (*SI Appendix*, Fig. S5*E*). At the same time, cells expressing CdvA_ΔE3B_ and CdvB failed to compact and individualize the two copies of their replicated genome (Fig. 2 *E* and *F*). These data imply that DNA segregation does not occur until after the assembly of a functional ESCRT-III division ring. Furthermore, in this experiment, we observed a small number of cells expressing CdvA_ΔE3B_ that slipped through the division arrest. These contained a single mass of DNA on one side of the partially constricted defective division ring (Fig. 2*E*)—reinforcing the idea that DNA segregation requires the assembly of a fully functional division ring. Similarly, in live imaging experiments, cells expressing CdvA_ΔE3B_ remained stuck in a state with highly mobile DNA for up to 2 h, as if unable to compact and segregate their DNA and divide (Movies S3–S5).

Next, to determine how cells align the axis of DNA separation relative to the future plane of the cytokinetic furrow, we analyzed live imaging data to generate a set of line profiles perpendicular to the future division plane which we could then use to compare dynamic changes in the DNA and membrane signal. These two-color kymographs revealed that DNA segregation occurs around the future plane of membrane constriction (Fig. 2*G*). By measuring membrane signal intensity across the whole cell during the earliest phase of constriction, we were then able to determine the position of the future cytokinetic furrow, which we could use as a landmark to compare with the position at which the gap between the new condensing nucleoids first becomes visible, a few minutes earlier. While limits in the resolution of light microscopy made it difficult to determine with high confidence whether the DNA segregates away from the geometric cell center or away from the site of the future furrow in cells that divide relatively symmetrically (Fig. 2*G*), in rarer asymmetric cell divisions it was clear that the DNA is cleared from a site that prefigures the division plane rather than being aligned with the middle of the cell (Fig. 2*H*). Taken together, these results show that maturation of the division ring through the recruitment of ESCRT-III proteins is a prerequisite for passage through the regulatory decision point that triggers the onset of DNA segregation and cytokinesis.

### SegA and SegB Regulate DNA Compaction.

Having shown that assembly of the ESCRT-III division ring is a prerequisite for DNA segregation, we next investigated the machinery involved in DNA compaction and individualization itself. As potential regulators, we turned our attention to the proteins SegA and SegB, since these proteins are expressed at the same time as ESCRT-III proteins in preparation for cell division (42), and have been suggested to bind DNA to drive chromosome segregation in *Sulfolobus* (34, 35).

**Fig. 3.**
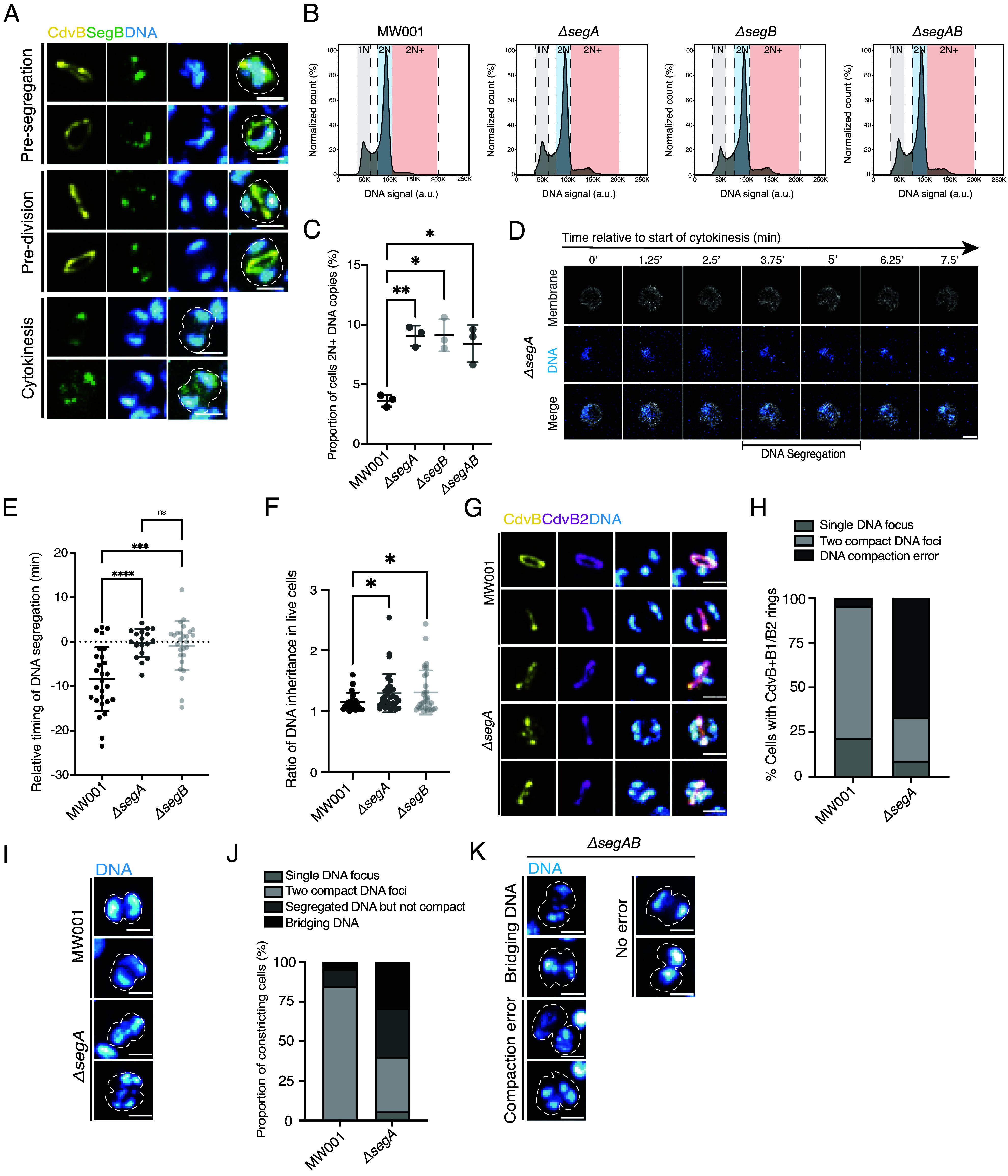
SegAB temporally coordinates DNA compaction with cell division. (*A*) Representative immunofluorescence images of control DSM639 cells stained with ESCRT-III protein CdvB (yellow), DAPI (blue), and SegB (green) in different stages of division phase; pre-DNA segregation, post-DNA segregation, and cytokinesis. All scale bars, 1 μm. (*B*) Representative flow cytograms of control MW001 cells and gene deletion strains *ΔsegA, ΔsegB,* and *ΔsegAB* showing a slight increase in the 2N+ cell population, n = 0.25 × 10^6^ (N = 3 biological replicates). (*C*) Quantification from flow cytograms of proportion of cells in populations of MW001, *ΔsegA, ΔsegB,* and *ΔsegAB* that have 2N+ copies of DNA. Significance values were derived using Welsch’s *t* test (N = 3 biological replicates). Mean plotted with error bars denoting ± SD. MW001 vs. *segA, P-*value *=* 0.0019, MW001 vs. *ΔsegB, P-*value = 0.0113, MW001 vs. *ΔsegAB, P-*value = 0.0250. (*D*) Montage of dividing *ΔsegA* cell from the first frame of constriction in live imaging where cells segregate their DNA asymmetrically after the onset of cytokinesis. (*E*) Quantification of timing of DNA segregation relative to timing of the first frame of cell constriction in cytokinesis (0) in control MW001 (n = 27), *ΔsegA* (n = 18), and *ΔsegB* (n = 27) cells. *P*-values were derived by the Mann–Whitney *U* unpaired test (N = 3 biological replicates) MW001 vs. *ΔsegA, P-*value = 0.0001, MW001 vs. Δ*segB P*-value = 0.0002. (*F*) Quantification of DNA asymmetry in inheriting daughter cells from live imaging of MW001 (n = 26), *ΔsegA* (n = 34), and *ΔsegB* (n = 32). *P*-values were derived by Welch’s *t* test. MW001 vs. *ΔsegA, P-*value = 0.0240, MW001 vs. *ΔsegB, P-*value = 0.0304. Mean plotted with error bars denoting ± SD (*G*) Control MW001 and *ΔsegA* cells with ESCRT-III rings in division phase stained with CdvB (yellow), CdvB2 (magenta), and DAPI (blue) to show DNA compaction. (*H*) Quantification of DNA compaction (compact into two foci, compaction error, or not yet segregated) in MW001 control (n = 46) and *ΔsegA* (n = 33) cells that have CdvB+CdvB1/B2 composite rings (N = 3 biological replicates). (*I*) MW001 control cells and *ΔsegA* cells fixed during cytokinesis and stained with DAPI (blue). (*J*) Quantification of DNA state in these constricting cells (Single DNA focus, compact into two foci, compaction error or bridging DNA) (MW001 n = 46, *ΔsegA* cells n = 52) (N = 3 biological replicates). (*K*) Constricting Δ*segAB* cells with bridging DNA, compaction errors, or no errors. All scale bars, 1 μm.

To investigate their roles in DNA segregation and division we used antibodies raised against SegA and SegB (43) (kindly given to us by Arthur Charles-Orszag and Dyche Mullins) to visualize the localization of the endogenous protein in cells in different stages of cell division (Fig. 3*A*). In wildtype cells that had not yet undergone DNA segregation, SegB was seen localizing in puncta on the DNA. As the DNA became more compact, these structures tended to coalesce into two well-defined loci, which remained in place throughout DNA segregation and cytokinesis, as expected if SegB binds SegS sites as proposed (43, 44). At the same time, many cells contained additional foci that were less obviously associated with the main nucleoid mass. We also used immunolocalization to investigate localization of SegA (43) in a similar manner. We visualized cells in different stages of cell division based on the DNA localization and found that SegA, like SegB, was also localized on the DNA both before and after DNA segregation (*SI Appendix*, Fig. S9). These data are consistent with the possibility that SegA and SegB play a role in DNA compaction and segregation at both early and late stages of division (Fig. 3*A*).

To test this idea and determine whether SegA and SegB play an active role in DNA segregation, we generated knockout strains for SegA (*ΔsegA*) and SegB (*ΔsegB*) as well as a double knockout strain lacking both proteins (*ΔsegAB*)—all of which were viable and grow well. By flow cytometry analysis, the loss of SegA and/or SegB led to a small, but statistically significant increase in the number of cells with more than two copies of the genome relative to the control (Fig. 3 *B* and *C*).

To determine the origin of this modest division phenotype, we imaged *ΔsegA* and *ΔsegB* cells live (Fig. 3 *D*–*F* and *SI Appendix*, Fig. S6). In interphase, mutant cells appeared similar to the MW001 control. However, while DNA became mobile as *ΔsegA* and *ΔsegB* cells entered division, as it does in wildtype cells, there was a profound change in the transition from mobility to DNA segregation in the mutants. Most strikingly, many cells in the deletion strains underwent DNA segregation after the onset of membrane constriction—something rarely seen in the corresponding MW001 control (Fig. 3*E* and *SI Appendix*, Fig. S6). Despite this, most *ΔsegA* and *ΔsegB* cells were still able to visibly compact and separate their DNA into two foci by late stages of cytokinesis. In line with this result, when the sum intensity of DNA in each daughter cell during cytokinesis was quantified, we saw only a marginal increase in the average asymmetry of DNA inheritance following the onset of cell division in nascent mutant daughter cells as compared to the MW001 control (Fig. 3*F*). Taken together these data show that while SegA and SegB play an important role in the temporal coordination of DNA segregation and cell division, backup systems likely aid DNA segregation in their absence.

To investigate whether SegA and SegB play a role in DNA compaction itself, we investigated DNA organization at higher resolution in mutant cells that had been fixed and stained using confocal microscopy. We focused on DNA organization in cells possessing a full ESCRT-III ring, as determined by the presence of CdvB and CdvB1 or B2, at a time when DNA segregation would normally be complete (Fig. 2). In contrast to the control, *ΔsegA, ΔsegB,* and *ΔsegAB* cells frequently failed to compact their DNA into two foci (Fig. 3 *G* and *H* and *SI Appendix*, Fig. S7). Instead, the DNA was seen in bright spots that were connected by thin linear segments.

This phenotype persisted in *ΔsegA* and *ΔsegB* cells that had been fixed mid-constriction. In these cells, DNA was often seen bridging the cytokinetic furrow—a phenotype that was extremely rare in dividing MW001 control cells (Fig. 3 *I* and *J*). Although this phenotype was striking, a subset of dividing cells in all three deletion strains correctly compacted and segregated their DNA. Again, these data imply the existence of additional machinery that acts together with SegA and SegB to aid DNA segregation (Fig. 3*K* and *SI Appendix*, Fig. S7). Furthermore, while the overexpression of the *SegAB* cassette under the control of an inducible promoter generated proteins that localized correctly to the DNA, their expression was not sufficient to induce compaction or DNA segregation (*SI Appendix*, Fig. S8). Taken together these results suggest that while SegA and SegB are not sufficient to induce premature DNA compaction and individualization, and are not essential for DNA segregation, they play a significant role in the temporal coupling of DNA compaction and separation with cell division in *Sulfolobus*.

## Discussion

**Fig. 4.**
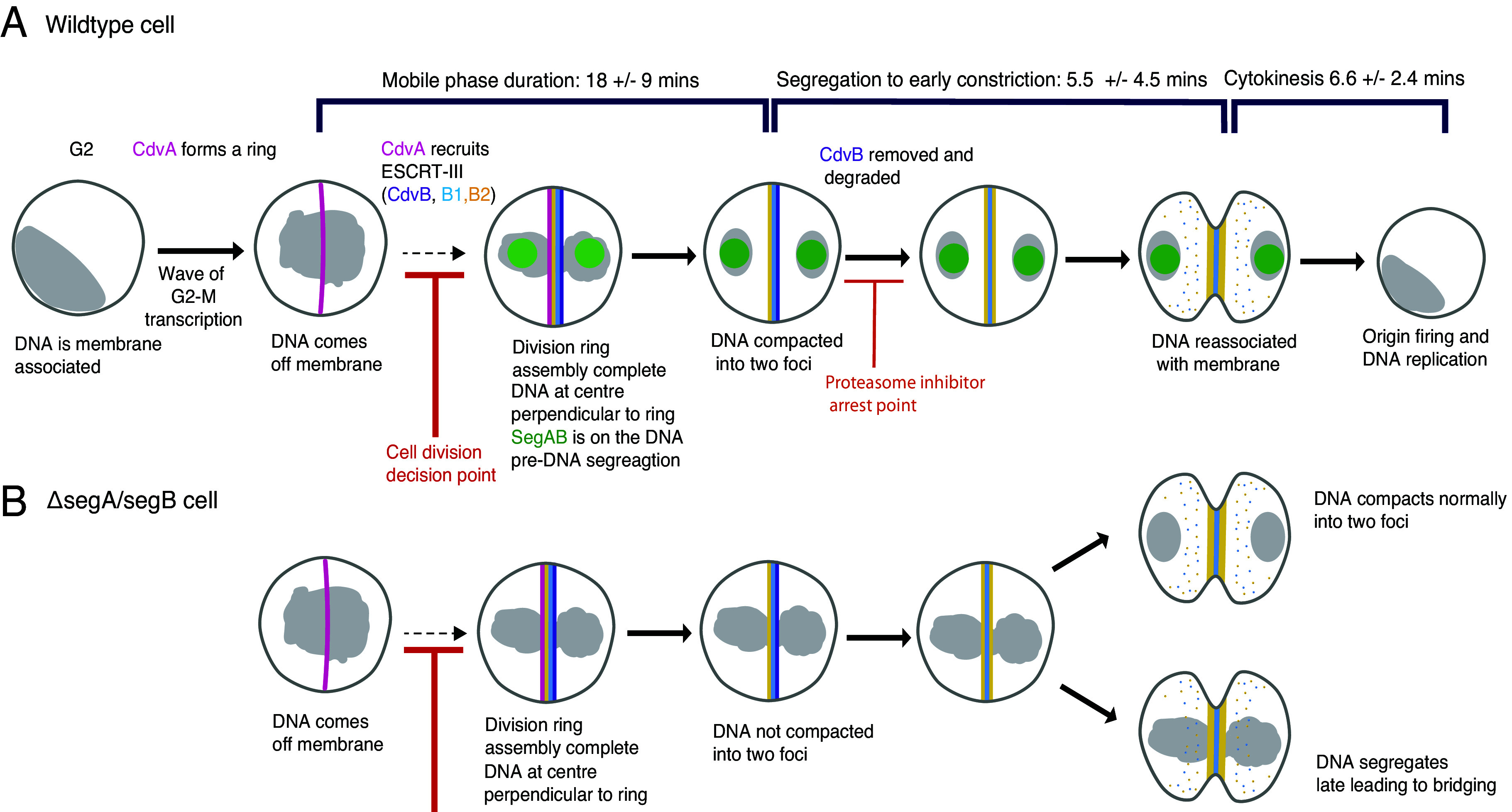
Schematic diagram of *Sulfolobus* cell division control. (*A*) Figure depicting a model for the regulation of *Sulfolobus* cell division. Cells undergo a wave of transcription at the G2-M transition leading to the coordinated expression of division and segregation proteins. CdvA forms a medial division ring before the expression of ESCRT-III, but this is not sufficient to induce DNA segregation. CdvA recruits ESCRT-III proteins CdvB and CdvB1 and B2 to the division ring. Perturbing full ESCRT-III ring assembly at this stage blocks DNA segregation, cell division, and cell cycle progression (which includes CdvB degradation). If cells pass through this regulatory checkpoint, however, the two nucleoids segregate via compaction in a process that requires SegA and SegB (and other undescribed factors). In wildtype cells expressing SegA and SegB, nucleoid segregation occurs rapidly and is complete before the onset of cytokinesis. In parallel, the CdvB ring is removed and CdvB is degraded to allow CdvB1 and CdvB2 rings to constrict, driving cell division. At the same time, other targets of the proteasome are removed to trigger DNA relicensing and entry into G1 and S phase. (*B*) While many *segA/segB* knockout cells divide successfully, most exhibit DNA segregation and compaction errors. The loss of SegA or SegB also leads to the accumulation of a subset of dividing cells with bridging DNA.

In this study, we use *S. acidocaldarius* as a model to characterize the series of events that ensure the correct partitioning of a single copy of the genome into two daughter cells in an archaeal relative of eukaryotes. This work reveals the existence of a regulatory decision point in the archaeal cell cycle that *Sulfolobus* cells use to coordinate DNA segregation with cytokinesis (Fig. 4). This functions to achieve the same goal as the spindle checkpoint in human cells and the spindle positioning checkpoint in yeast (45, 46)—to ensure cells are ready before they commit to division and reenter G1 of the following cell cycle.

In *Sulfolobus* cells, as in human cells, passage through this control point is associated with the transition from a single DNA mass to two spatially separated masses. After this point, cells are committed to division and entry into G1. This implies that the action of a pre-DNA segregation regulatory control point may be a general feature of cell cycle control in many systems and was likely in existence in archaea prior to the advent of regulatory oscillations in the levels of CDK-cyclin activity. This does not mean that one should necessarily expect any of the machinery involved in this predivision checkpoint to be conserved from archaea to eukaryotes, since it is possible that using a checkpoint to regulate entry into division prior to DNA segregation is simply an elegant solution to the challenges faced when trying to coordinate DNA segregation and division that has evolved independently multiple times. In the future, it will be interesting to know whether similar checkpoints exist in the Asgard archaea [also referred to as *Asgardarchaeota* (47) and *Prometheoarchaeum* (48)], which are relatives of *Sulfolobus* (49), and the closest known archaeal relatives of eukaryotes (50).

As we show in this paper, the complex series of events that accompany division in *Sulfolobus* begin up to half an hour before division, when the duplicated genome loses its association with the bounding cell membrane—similar to the way DNA comes away from the nuclear envelope in a eukaryotic cell entering mitosis (51). After membrane detachment, the duplicated genome becomes both diffuse and highly mobile. In fact, the localization of genomic DNA appears to change within the 15 s that separate frames in our movies (Movies S1 and S2). While this mobile phase lasts a variable amount of time, it comes to an end with rapid DNA compaction.

The computational model we developed to test the likely role of these observed DNA dynamics in DNA segregation shows that entropic forces can help disentangle DNA polymers in spherical cells, like *Sulfolobus*, even though these forces are weaker than in cells having an elongated shape (41). This shows that entropy could play a more general role in genome segregation in cells with different shapes, including in spherical cells. In this light, the “mobile phase” of division likely facilitates the entropic demixing and individualization of the two copies of the *Sulfolobus* genome. This mobile phase appears to come to a sudden end as the DNA compacts. Our simulations show that this rapid phase of compaction can aid the separation of the DNA into two spatially separate masses in two ways: It both locks in the effects of entropic separation to aid DNA segregation (hence the need for speed), and reduces DNA mobility. In the model, efficient DNA segregation is also facilitated by the reassociation of the DNA with the membrane at cell poles—something that is usually complete by late stages of cytokinesis. Although this analysis suggests ways in which the dynamic changes in DNA–membrane association (high in G2, low in division, high during cytokinesis) and DNA compaction (high in G2, low pre-DNA segregation, and high predivision) observed in cells likely contribute to DNA segregation, it is clear from the model that other ingredients need to be added to enable robust and complete DNA individualization, as discussed below.

At the same time as these dramatic changes in DNA organization are underway, cells assemble a medial cytokinetic ring (Fig. 4). At the stage in the cell cycle when the DNA is mobile, our data from fixed cells suggest that the template protein CdvA begins forming a ring at the future site of cytokinesis. This CdvA ring then recruits the ESCRT-III homologue, CdvB (which lies downstream of CdvA in the CdvABC operon), likely through an interaction between the broken winged helix domain of CdvB and CdvA’s E3B tail (27). This CdvAB ring in turn recruits the contractile ESCRT-III homologues CdvB1 and CdvB2, leading to the formation of a complete composite ring that, once activated, contracts to cut cells into two (19).

Crucially, our data show that the assembly of this mature composite division ring sets the stage for the subsequent events (Fig. 4). Thus, completion of the ring performs several functions: i) It enables cytokinesis, ii) it is required to trigger the compaction of the DNA into two individualized masses, iii) and it provides a cue that is used to ensure that the axis of DNA segregation is perpendicular to the division axis. Precisely how cells monitor completion of the division ring and use this information to direct DNA segregation is a fascinating question for the future.

Once the checkpoint has been satisfied and DNA segregation has begun, CdvB is removed from the predivision ring by Vps4 (24), allowing for its subsequent degradation by the proteasome - whose activity is also required during division for DNA replication in the following cycle (19, 52). Interestingly, we saw no evidence to support the existence of an additional checkpoint that monitors ring constriction or DNA segregation itself, since DNA segregation and DNA replication appear unperturbed in cells expressing a dominant negative Vps4 (24). Furthermore, progression through cytokinesis occurs on schedule in cells lacking SegA and SegB. Thus, as with the spindle checkpoint in human cells (45), *Sulfolobus* cells seem to have invested all control in a single decision point prior to DNA segregation that must be passed for cells to progress into the next phase of the cell cycle. Once this has been passed, cells in both systems still initiate cytokinesis even if they fail to segregate their DNA (53).

While we hope that future work will identify the machinery used to assess ring completion and transmit this information to different elements of the downstream machinery, in this paper, we show that SegA and SegB play a critical role in the temporal coordination of ring completion and DNA segregation. We show that these two proteins, which sit in the same operon, are required together for the timely compaction of DNA into two spatially separate masses following completion of the division ring. In their absence, cytokinesis is initiated prior to DNA segregation, leading to the accumulation of cells with bridging DNA. Nevertheless, while SegA and SegB perform an important role in DNA compaction and segregation (43), the fact that cells are still able to complete DNA segregation in their absence [something also observed in a parallel study by Charles-Orszag et al. (43)] points to the existence of unknown molecular players that also contribute to the process. Given the importance of cell division for lineage survival, it is not surprising to find that the system is robust to perturbation. The other molecules involved may include a set of recently identified DNA binding proteins (54, 55). It is worth noting, however, that while almost all bacteria and eukaryotes rely on SMC-like proteins to individualize intertwined DNA strands by scanning the DNA in *cis* and generating loops (sometimes in partnership with bacterial ParA and ParB proteins), it is not clear whether a similar system operates in *Sulfolobus*. The only SMC protein thus-far identified in *Sulfolobales,* called Coalescin, is more similar to Rad50 than it is to Condensin or Cohesin (31). Furthermore, the timing of Coalescin expression is significantly later than that of ESCRT-III and SegAB proteins (18), suggesting a role for the protein in S or G2 phase rather than in DNA segregation or cell division. Given the need for additional systems to facilitate high fidelity division in our model, we think it likely that there are non-SMC proteins that operate in an analogous way to aid chromosome individualization in *Sulfolobus*.

Using our computational model of *Sulfolobus* division to test this idea, we introduced a signal that spreads along individual chromosomes from one site to induce DNA compaction in *cis*. This addition greatly improved the efficacy of DNA segregation when compared to the effects of global compaction (*SI Appendix*, Fig. S3*G*). While it is not clear what machinery might perform such a role in cells, it is possible that SegA and SegB drive DNA segregation in this manner, for example by generating loops via the SegA-dependent physical association of neighboring SegB-bound regions of the genome (44). In this case, to achieve the spacing between segregated nucleoids we observe via light microscopy, it would likely be important to orient the initiating ParS sites relative to the division plane before initiating spreading compaction. Whether or not this is how compaction works in *Sulfolobales* is a question we and others will need to resolve in the future.

## Materials and Methods

### Strains, Culture Media, and Growth Conditions.

*S. acidocaldarius* strains DSM639 (wildtype) MW001-CdvA (MW001 with the full length and ΔE3B domain truncation), MW001-SegAB (MW001 with the SegAB overexpression plasmid), and MW001-EV (MW001 with the empty vector plasmid) were grown at 75 °C, pH 3.0 to 3.5, in Brock medium supplemented with 0.1% w/v NZ-amine and 0.2% w/v sucrose. The mutant *S. acidocaldarius* strains MW001 (uracil auxotroph, genetic background), MW001-Δ*segA* (MW001 with *segA* gene deletion), MW001-Δ*segB* (MW001 with *SegB* gene deletion), MW001-Δ*segAB* (MW001 with *SegAB* gene deletion) were supplemented with 4 μg/mL uracil. The optical density of liquid cell cultures was maintained at levels corresponding to exponential growth, OD_600_ values between 0.100 and 0.400, for between 2 to 4 d prior to experiments and samples were only taken within this range. Cells were fixed by stepwise addition of absolute ethanol until a concentration of 70%. Cells stained with SegA antibody were fixed with 4% formaldehyde and treated with 0.01% SDS for 20 min.

### Cell Synchronization.

*S. acidocaldarius* cultures were arrested by treatment with acetic acid (final concentration 3 mM) for 4 h. After arrest, cells were washed three times in fresh Brock medium to remove the acetic acid and released into fresh media to reenter the cell cycle. Cells were then fixed as previously described at different time points postrelease.

### Molecular Genetics.

Deletion mutants were constructed using the pSVA406 vector as described previously (36, 56). Briefly, the upstream and downstream regions of a gene of interest was amplified via PCR and cloned into pSVA406 through restriction digest. Positive clones were selected through analytical digestion, methylated and purified via miniprep for transformation into electrocompetent *S. acidocaldarius* cells. Positive strains containing the integrated inserts were next restreaked onto Gelrite-Brock plates supplemented with 4 μg/mL uracil and 100 μg/mL 5-fluoroorotic acid (Zymo Research, P9001-1). Colonies obtained were then selected, grown, and screened for the deletion of the gene of interest through genomic DNA extraction and genotyping of the overlapping regions of the locus of interest. Positive clones were frozen in Brock medium containing 50% glycerol (v/v) and stored at −70 °C.

The SegAB deletion mutant was constructed by the deletion of the entire operon from the start codon of SegA to the stop codon of SegB inclusive. Due to the four nucleotide overlap between the SegA and SegB genes, the single Seg mutants were constructed by deletion of the gene of interest without affecting the neighboring gene: The SegA deletion mutant was constructed by the deletion of the gene from the start codon to before the start codon of SegB; the SegB deletion mutant was constructed by the deletion of the gene from after the stop codon of SegA to the stop codon of SegB.

To generate the overexpression plasmid of SegAB with C-terminal HA tag, Saci_0203+Saci_0204 (uniport accession number Q4JC57 and Q4JC56) was PCR amplified from the genomic DNA of *S. acidocaldarius* DSM639 using the primer pair: 5′- AATccatggctATGATAATCACTGTCATCAA-3′ and 3′CGCctcgagACTCTTTTTACTCTCTAATG-5′ and cloned into the plasmid vector containing arabinose-inducible promoter (pSVAaraFX- HA). The PCR product was purified by the Monarch DNA cleanup kit (New England Biolabs) and the vector was digested with restriction enzymes NcoI and XhoI followed by gel extraction. The linearized vector and purified PCR product were assembled by Gibson assembly using the overlapping sequence. The sequence of the cloned plasmid (pSVAaraFX-SegAB-HA) was verified by Sanger sequencing.

To generate the overexpression plasmid of CdvA_ΔE3B_ with C-terminal HA tag, 1 to 220 aa of saci_1374 (uniport accession number Q4J923) was PCR amplified from the genomic DNA of *S. acidocaldarius* DSM639 using the primer pair: 5′-taataattgataagcgtcttacttatcataccATGGGCATTCCGGTTGA-3′ and 5′-tacgcgtagtccggaacgtcatacgggtactcgagCTCGTTCTTATTTGACTGTTCTGTTG-3′ and cloned into the plasmid vector containing arabinose-inducible promoter (pSVAaraFX- HA). The PCR product was purified by the Monarch DNA cleanup kit (New England Biolabs) followed by gel extraction. The linearized vector and purified PCR product were assembled by Gibson assembly using the overlapping sequence flanking the CdvA_ΔE3B_ (lower case region of the primers above). The sequence of the cloned plasmid (pSVAaraFX-CdvA_ΔE3B_-HA) was verified by Sanger sequencing.

### Live Cell Imaging of *S. acidocaldarius*.

Live cell imaging was performed using the Sulfoscope set-up as described (37) with additional hardware modifications detailed (24). Briefly, Attofluor chambers (Invitrogen A7816) were assembled with 25 mm coverslips and filled with 300 µL Brock medium (hereafter referred to as BNS). The medium was allowed to dry onto the surface of the coverslip at 75 °C before the chambers were washed thoroughly with BNS and placed into the preheated Sulfoscope and allowed to equilibrate to 75 °C. 5 mL of *S. acidocaldarius* cell culture at an optical density at 600 nm (OD_600nm_) of 0.15 to 0.3 was kept at 75 °C and stained with CellMask Deep Red Plasma Membrane (Invitrogen C10046; 1:5,000). 400 µL of the stained cell suspension was transferred to the preheated Attofluor chamber, making sure to avoid cooling of the chamber or the cells. Cells were immobilized using semisolid Gelrite pads (0.6% Gelrite, 0.5× BNS pH 5, 20 mM CaCl_2_). Gelrite pads were prepared by mixing thoroughly preheated BNS (pH 5) and 1.2% Gelrite in a 1:1 ratio. To set, 15 mL of the molten Gelrite BNS solution was added to 9 cm plastic petri dishes and allowed to set at room temperature (~5 min). To prepare the immobilization pads, half-moon shapes were cut from the plate with a 7 mm diameter circle punch and placed onto 13-mm circular coverslips. Immediately before imaging pads were incubated at 75 °C for 5 min in a bead bath until they equilibrated to the imaging temperature as well as began to dry causing the edges of the pad to curve downward. Upon adding the cell suspension, the preheated immobilization pad was placed such that the concave edge of the pad was in the center of the chamber. Cells were imaged at this concave edge where diffusion is limited but cells are not subjected to mechanical stress by the pad. Images were acquired on a Nikon Eclipse Ti2 inverted microscope equipped with a Yokogawa SoRa scanner unit with an additional 2.8× magnification and Prime 95B scientific complementary metal-oxide semiconductor (sCMOS) camera (Photometrics). Imaging was performed with a 60× oil immersion objective (Plan Apo 60×/1.45, Nikon) using a custom formulated immersion oil for high temperature imaging (maximum refractive index matching at 70 °C, n = 1.515 ± 0.0005; Cargille Laboratories). Images were acquired at a total magnification of 168× with 15-ms exposure time and 10% laser power at intervals of 15 s for 2.5 h or until any cell death was observed. XY drift was corrected after acquisition using the ImageJ plugin StackReg (57).

### Live Imaging Quantification.

As a first step, time-lapse microscopy image sequences were manually cropped using Fiji to isolate regions of interest containing individual cells. Each cropped sequence was subsequently processed via a custom Python script to segment cellular and DNA components, quantify apparent motion, and visualize the results. Prior to segmentation, images underwent preprocessing, consisting of a spatiotemporal Gaussian filter and deblurring via 10 iterations of the Richardson-Lucy algorithm (58, 59). Cellular segmentation and tracking were performed using the “cyto2” model implemented in Cellpose (60), employing a diameter parameter of 22 and utilizing both available channels. In instances where the resulting masks were null, a watershed segmentation approach was employed as an alternative. The centroid of the resulting mask was then tracked using nearest-neighbor assignment, with other segmented regions being discarded. Apparent motion, or optical flow, was calculated for each channel using the Lucas–Kanade algorithm (61) as implemented in scikit-image (62). DNA signal segmentation was achieved using a difference of Gaussians filter, followed by temporal tracking using the Crocker and Grier algorithm (63) as implemented in trackpy (64). Processed images, segmentation masks, and calculated flow fields were then stored in HDF5 format. Subsequently, frame difference, momentum, and momentum divergence were computed and visualized as streamlines. Furthermore, the mean of the magnitude of each of these three derived quantities, calculated within the segmented cellular regions, was determined at each time point to quantitatively assess DNA motion over time.

### Immunolabeling.

Fixed cells were washed and rehydrated in phosphate buffered saline (PBS)-TA (PBS supplemented with 0.2% Tween20 and 3% bovine serum albumin) before incubation overnight at 25 °C and 400 rpm agitation with primary antibodies (*SI Appendix*, Table S1). Conjugated secondary antibodies were used for detection of target proteins (*SI Appendix*, Table S2) by staining for 2 to 3 h at 25 °C and 400 rpm agitation. The S-layer was stained by incubating the rehydrated sample with 200 μg/mL Concanavalin A conjugated to Alexa Fluor 647 (ThermoFisher, C21421) for 2 to 3 h. DNA was visualized by the addition of 1 μg/mL DAPI (Thermo Fisher Scientific, 62248) to samples after secondary antibody incubation. For spinning disc microscopy, Lab-Tek chambered slides (Thermo Fisher Scientific, 177437PK) were coated with 2% polyethyleneimine for 30 min at 37 °C. Chambers were washed with Milli-Q water before stained cell suspension was added and spun down for 1 h at 750 relative centrifugal force.

### Spinning Disc Microscopy.

Cells were imaged in Lab-Tek chambered coverslip using a Nikon Eclipse Ti2 inverted microscope equipped with a Yokogawa SoRa scanner unit and Prime 95B sCMOS camera (Photometrics). Images were acquired with a 100× oil immersion objective (Apo TIRF 100×/1.49, Nikon) using immersion oil (immersion oil type F2, Nikon). A total magnification of 280× was achieved using the 2.8× magnification lens in the SoRa unit. Images were acquired with 200-ms exposure time for labeled proteins and 500-ms exposure time for DNA stains, with laser power set to 20%. *z*-axis data were acquired using 10 captures with a 0.18 to 0.22-μm step.

### Flow Cytometry.

DNA was labeled with 2 μM Hoechst for flow cytometry. Cells were gated by DNA staining (UV excitation). Laser excitation wavelengths of 355, 488, 561, and 633 nm were used in conjunction with the emission filters 450/50, 530/30, 586/15, and 670/14, respectively. Flow cytometry analysis was performed on BD Biosciences LSRFortessa. Side scatter and forward scatter was recorded. Analysis was performed using FlowJo v10.8.1.

### Statistical Analysis.

All statistical analysis was performed in Microsoft Excel or GraphPad Prism 10 software. Significance was defined as *P* ≤ 0.05. Significance levels used were ^*^P ≤ 0.05, ^**^P ≤ 0.01, ^***^P ≤ 0.001 and ^****^P ≤ 0.0001. Exact statistical tests are reported in the figure legends.

## Supplementary Material

Appendix 01 (PDF)

Movie S1. Live cell imaging examples of wildtype cells (DSM639) undergoing DNA segregation and cell division (Cellmask membrane stain in grey and DNA in cyan).

Movie S2. Live cell imaging examples of wildtype cells (DSM639) undergoing DNA segregation and cell division (Cellmask membrane stain in grey and DNA in cyan).

Movie S3. Live cell imaging examples of CdvA_ΔE3B_ mutants arrested in DNA mobile phase with no progression to DNA segregation or cell division (Cellmask membrane stain in grey and DNA in cyan).

Movie S4. Live cell imaging examples of CdvA_ΔE3B_ mutants arrested in DNA mobile phase with no progression to DNA segregation or cell division (Cellmask membrane stain in grey and DNA in cyan).

Movie S5. Live cell imaging examples of CdvA_ΔE3B_ mutants arrested in DNA mobile phase with no progression to DNA segregation or cell division (Cellmask membrane stain in grey and DNA in cyan).

## Data Availability

Code used for analysis of the mobile phase in live cells is available on GitHub at https://github.com/jboulanger/dna-movement-sufolobus (65). The code associated with the computational model is freely available on GitHub at https://github.com/Saric-Group/archaea_chromosome_seg (66). All other data are included in the article and/or supporting information.
