# Supplementary material for: Temporal and spatial coordination of DNA segregation and cell division in an archaeon
Source: Proc Natl Acad Sci U S A. 2025 Oct 15;122(42):e2513939122. doi: 10.1073/pnas.2513939122 (PMC12557731; doi:10.1073/pnas.2513939122)
Supplement: Supplementary file 1 — Appendix 01 (PDF) [file pnas.2513939122.sapp.pdf]

Extended data for: Temporal and spatial coordination of DNA segregation and cell division in an archaeon

Authors: Joe Parham[1], Valerio Sorichetti[2], Alice Cezanne[1], Sherman Foo[1], Yin-Wei Kuo[1], Baukje Hoogenberg[1], Arthur Radoux-Mergault[1], Eloise Mawdesley[1], Lydia Daniels Gatward[1], Jerome Boulanger[1], Ulrike Schulze[1], Anđela Šarić[2] and Buzz Baum[1*]

Includes:

Supplementary Figures and legends (pages 2-14)
Supplementary Movie legends (page 15)
Supplementary tables (page 16)
Detailed description of the computational model (pages 17-24)
Supplementary References (pages 25-26)

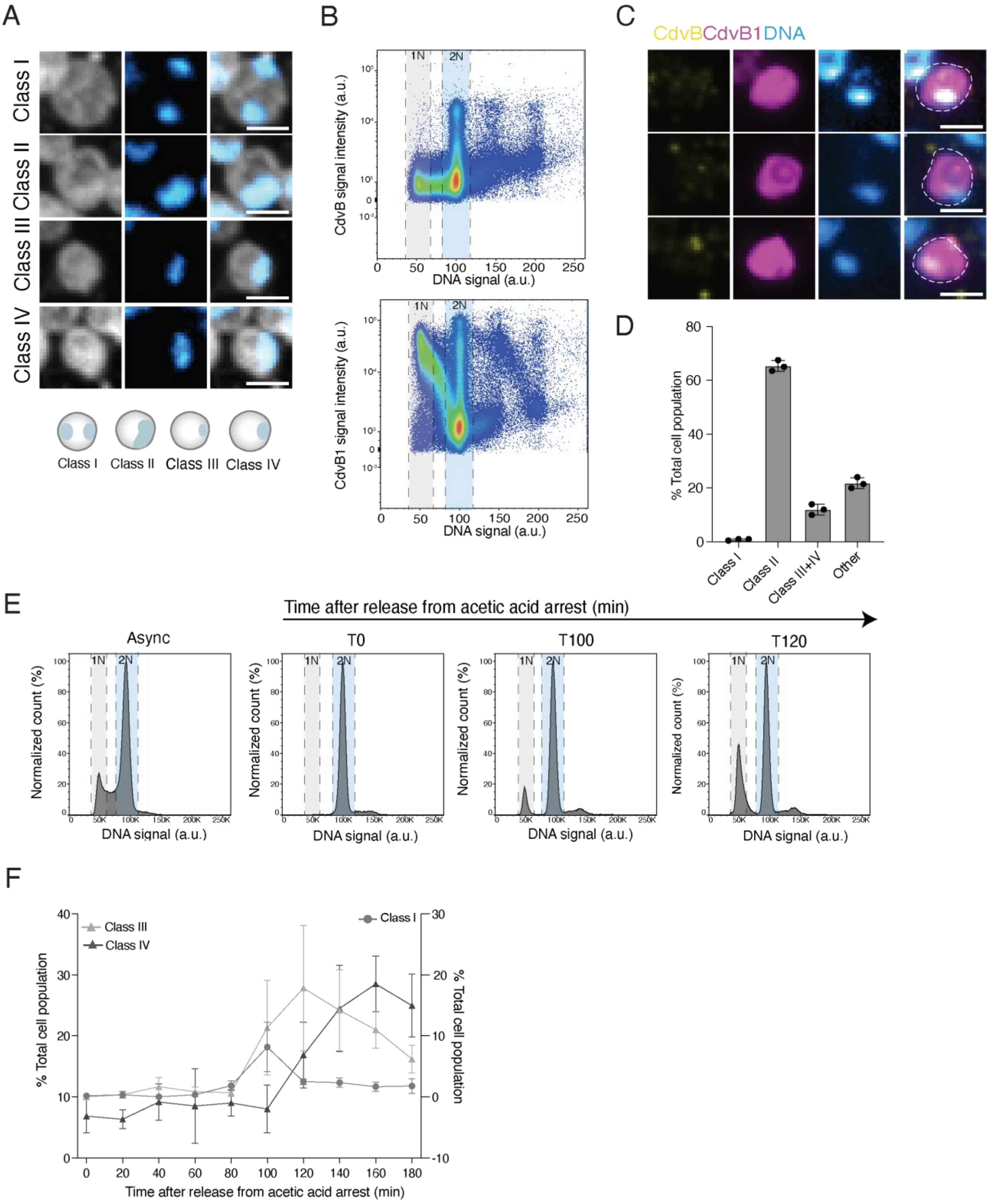
A
Class I
Class II
Class III
Class IV
Class I Class II Class III Class IV
B
1N
2N
CdvB signal intensity (a.u.)
DNA signal (a.u.)
CdvB1 signal intensity (a.u.)
0 50 100 150 200 250
C
CdvB CdvB1 DNA
D
% Total cell population
0 20 40 60 80
Class I
Class II
Class III+IV
Other
E
Time after release from acetic acid arrest (min)
Async
T0
T100
T120
Normalized count (%)
DNA signal (a.u.)
0 50K 100K 150K 200K 250K
F
Class III
Class IV
Class I
% Total cell population
0 10 20 30 40
-10 0 10 20 30
0 20 40 60 80 100 120 140 160 180
Time after release from acetic acid arrest (min)

Figure S1: DNA localisation is cell cycle regulated in *Sulfolobus*

(A) Panel shows representative images from spinning disc microscopy of wildtype *Sulfolobus* cells labelled with ConA to mark the cell outline (white) and DAPI to label DNA (blue) each of which is used to define a class of cells based on their DNA localisation. Scale bar =1µm. **(B)** Representative flow cytograms of wildtype cells from asynchronous cultures stained with CdvB and CdvB1 antibodies. Note that CdvB is absent from G1 cells. **(C)** Representative images of immunolabelled G1 cells that have low CdvB and high CdvB1 together with a single, small compact DNA nucleoid. Scale bar =1µm. **(D)** Quantification of the average proportions of major classes of DNA organisation in fixed cells from asynchronous cultures imaged by spinning disc microscopy defined based on the schematic on the left (n=600, N=3). “Other” represents cells with odd patterns of DNA organization that did not fit into the main categories. **(E)** Representative flow cytograms showing the DNA content of cells at different time points post-release from G2 arrest. **(F)** Proportion of cells with different DNA classes post-release from G2 arrest (n=600, N=3). Class I is plotted on the right Y axis and Classes III and IV are plotted on the left Y axis.

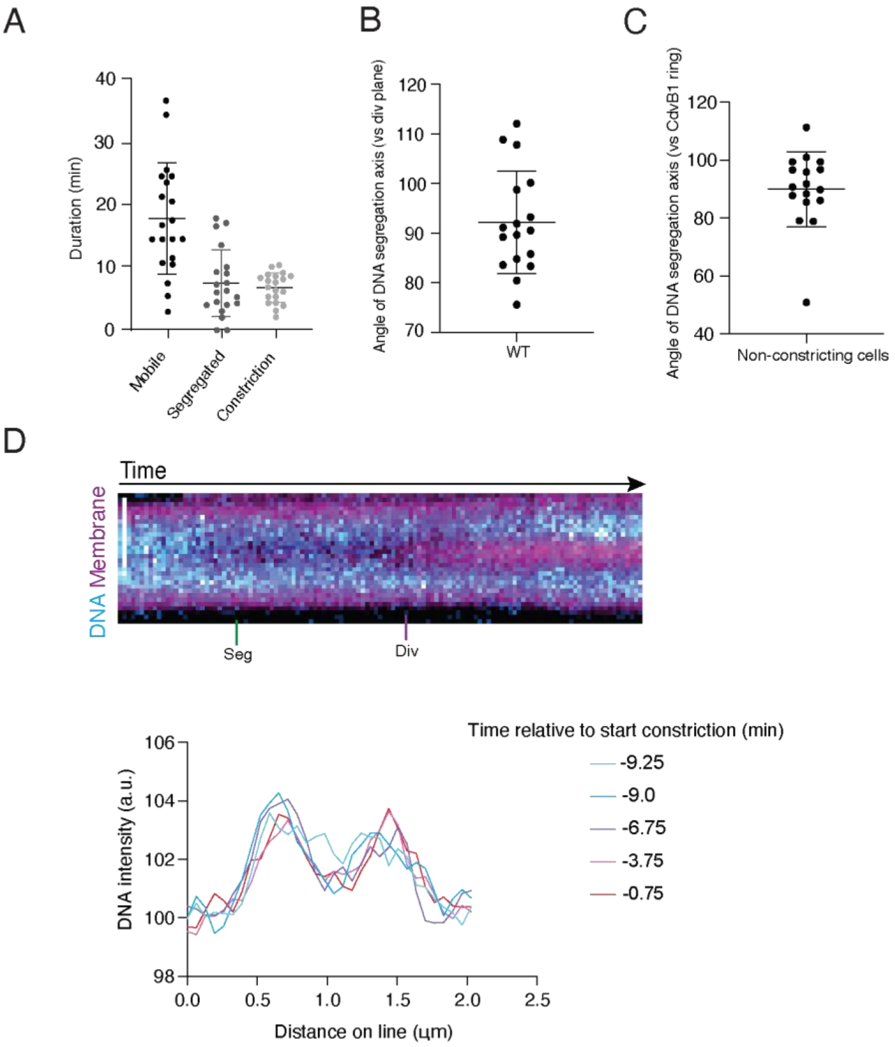
A
Duration (min)
40
30
20
10
0
Mobile
Segregated
Constriction
B
Angle of DNA segregation axis (vs div plane)
120
110
100
90
80
70
WT
C
Angle of DNA segregation axis (vs CdvB1 ring)
120
100
80
60
40
Non-constricting cells
D
Time
DNA Membrane
Seg
Div
Time relative to start constriction (min)
-9.25
-9.0
-6.75
-3.75
-0.75
DNA intensity (a.u.)
106
104
102
100
98
0.0
0.5
1.0
1.5
2.0
2.5
Distance on line (μm)

Figure S2: Live imaging reveals DNA dynamics during cell division

(A) Plot shows the duration of distinct phases of division (defined in Fig 1) in cells imaged live as they progress from G2 into division. These are defined as: the duration of the mobile phase; the time between DNA segregation and the onset of constriction; and the time between the onset of constriction and its end. (n=20). **(B)** Plot shows a quantification of the angle of DNA segregation axis relative to the plane of cell division (n=17) Mean plotted with error bars denoting ± std. **(C)** Plot of quantification of angle of DNA segregation relative to the position of the CdvB1 ring in fixed non-constricting cells (n=20). **(D)** Kymograph shows the DNA and membrane signal across a representative cell imaged live every 15 seconds as it enters division. The DNA segregation event marked with an arrow is rapid event (see arrow) and, after this has occurred, the two spatially separated masses of DNA do not move further apart**.** Scale bar =1μm. The corresponding line graph for the kymograph depicted in Fig S2D, showing the DNA intensity profile through a cell from the frame before DNA segregation (t = -9.25 minutes) to just before constriction (t = -0.75 minutes**)**.

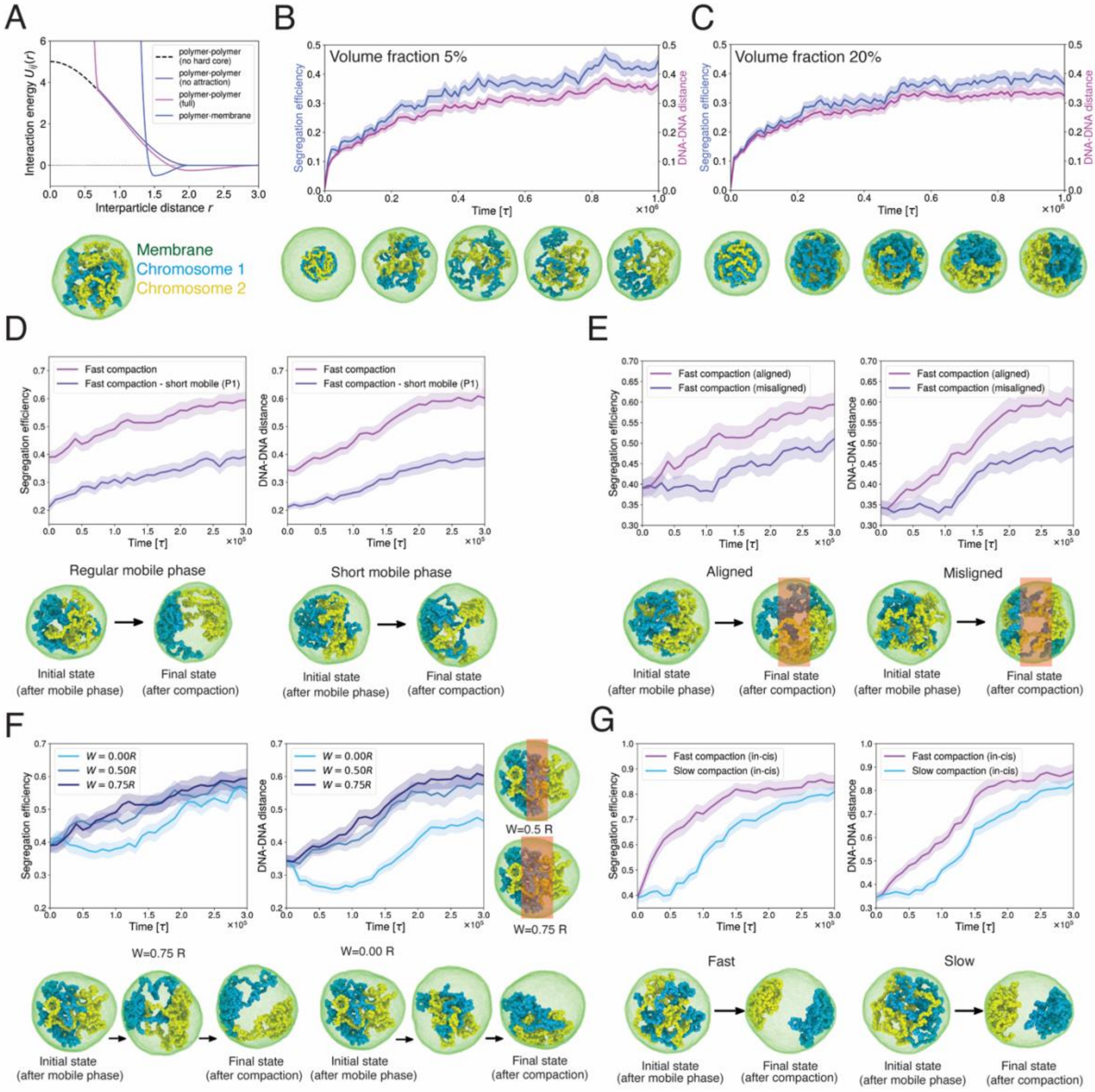
A
Interaction energy $U_{ij}(r)$
Interparticle distance r
polymer-polymer (no hard core)
polymer-polymer (no attraction)
polymer-polymer (full)
polymer-membrane
Membrane
Chromosome 1
Chromosome 2
B
Volume fraction 5%
Segregation efficiency
DNA-DNA distance
Time [τ]
C
Volume fraction 20%
Segregation efficiency
DNA-DNA distance
Time [τ]
D
Fast compaction
Fast compaction - short mobile (P1)
Segregation efficiency
DNA-DNA distance
Time [τ]
Regular mobile phase
Short mobile phase
Initial state (after mobile phase)
Final state (after compaction)
E
Fast compaction (aligned)
Fast compaction (misaligned)
Segregation efficiency
DNA-DNA distance
Time [τ]
Aligned
Misligned
Initial state (after mobile phase)
Final state (after compaction)
F
$W = 0.00R$
$W = 0.50R$
$W = 0.75R$
Segregation efficiency
DNA-DNA distance
Time [τ]
W=0.5 R
W=0.75 R
W=0.75 R
W=0.00 R
Initial state (after mobile phase)
Final state (after compaction)
G
Fast compaction (in-cis)
Slow compaction (in-cis)
Segregation efficiency
DNA-DNA distance
Time [τ]
Fast
Slow
Initial state (after mobile phase)
Final state (after compaction)

Figure S3: Extended simulation data

(A) Interaction potentials between polymer bead pairs and polymer-membrane pairs (see Eq 1-2). The potentials are shown ϵ_{pp}^{attr}= $0.25k_BT$ (full) and ϵ_{pp}^{attr}= 0 (no attraction) for the polymer-polymer interaction, and ϵ_{pm}^{attr}= $0.5k_BT$ for the polymer-membrane interaction. **(B-C)** Segregation efficiency and DNA-DNA distance in simulations of cells undergoing the mobile phase, for the chromosome volume fraction $\phi_p \approx 5\%$. **(B)** and $\phi_p \approx 20\%$. **(C)** The snapshots, which represent one of the simulated systems for each ϕ_p value, were taken at time intervals of $2.5\times10^5\tau$. **(D)** Graphs show segregation efficiency (left) and DNA-DNA distance (right) as measured in 50 simulations of cells undergoing fast DNA compaction. Comparison of compaction after a regular-duration mobile phase (duration $10^6\tau$) and after a short mobile phase (duration $10^5\tau$). The snapshots represent the initial state of compaction, together with compaction at the end of the mobile phase and in the final state, for two of the simulated systems after a regular (left) or short (right) mobile phase. **(E)** Graphs show segregation efficiency (left) and DNA-DNA distance (right) for 50 simulations of cells undergoing fast compaction. Here, we compare the case in which the zone in which compaction is inhibited is perpendicular to the axis of maximum segregation (aligned case) to the one in which there is no alignment (misaligned case). The snapshots represent the initial state of compaction at the end of the mobile phase and the final state reached, for one representative example of the simulated systems for both the aligned and misaligned cases. The red-shaded region defines the area in which compaction has been inhibited. **(F)** Graphs show segregation efficiency and DNA-DNA distance in 50 simulations of cells undergoing fast compaction for different widths W of the region in which compaction is inhibited, schematically represented on the right by the red-shaded regions. The snapshots, taken at intervals of $1.5\times10^5\tau$, show two simulated systems undergoing compaction for W= $0.75R$ and W= 0. The latter value corresponds to uniform compaction (G) Graphs show segregation efficiency (left) and DNA-DNA distance (right) in 50 simulations of cells undergoing fast and slow in *cis* compaction. The signal for compaction in *cis* propagates along the chromosome sequence, starting from two “initiator sites” located close to cell poles. The snapshots represent the initial state of compaction at the end of the mobile phase and the final state reached, for one representative example of the simulated systems for both fast and slow compaction. In all the snapshots, the cell membrane is coloured in green, while the two chromosomes are coloured in yellow and blue, respectively. For graphs Fig S3B-G, n=50, and the shaded area represents the standard error.

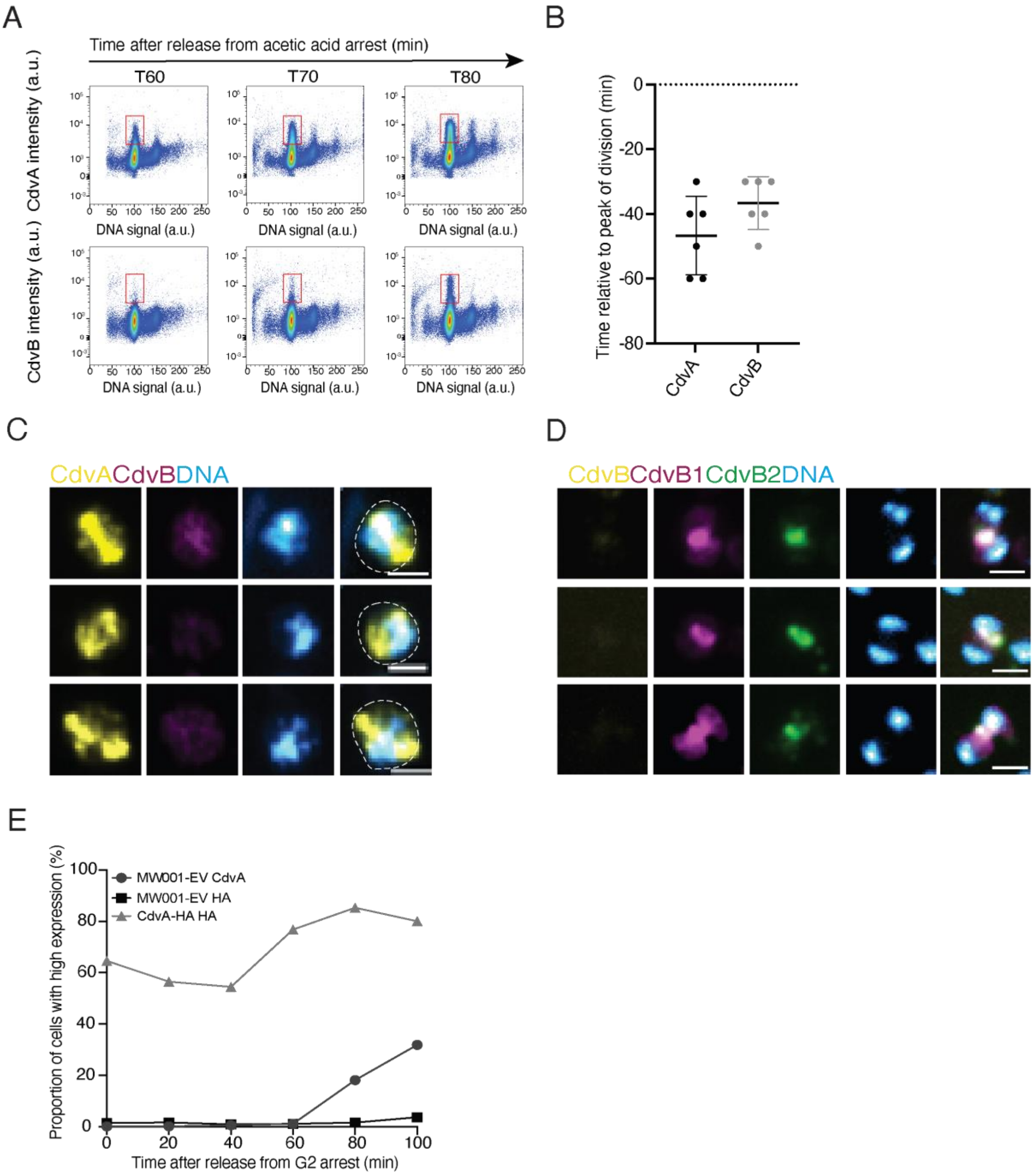
A
Time after release from acetic acid arrest (min)
T60
T70
T80
CdvA intensity (a.u.)
CdvB intensity (a.u.)
DNA signal (a.u.)
B
Time relative to peak of division (min)
0
-20
-40
-60
-80
CdvA
CdvB
C
CdvACdvBDNA
D
CdvBCdvB1CdvB2DNA
E
Proportion of cells with high expression (%)
MW001-EV CdvA
MW001-EV HA
CdvA-HA HA
Time after release from G2 arrest (min)

Figure S4: Ring states through cell division phase

(A) Representative flow cytograms of control (MW001) cells at sequential time points post-release from a G2 acetic acid arrest that have been fixed and stained for CdvA and CdvB. **(B)** Plot shows first observable expression of CdvA and CdvB following the release of wildtype (DSM639) cells from a G2 arrest as quantified from cytograms relative to the peak of division (t=0), N=6. **(C)** Representative images showing DNA in DSM639 cells with ring-like CdvA structures before the accumulation of ESCRT-III polymers. **(D)** Representative images of DNA in DSM639 cells in late division as measured by the presence of contracting CdvB1 and CdvB2 rings following the removal of CdvB from the ring. **(E)** Quantification of CdvA-HA induction in an early induced overexpression during an acetic acid arrest. Scale bars =1μm.

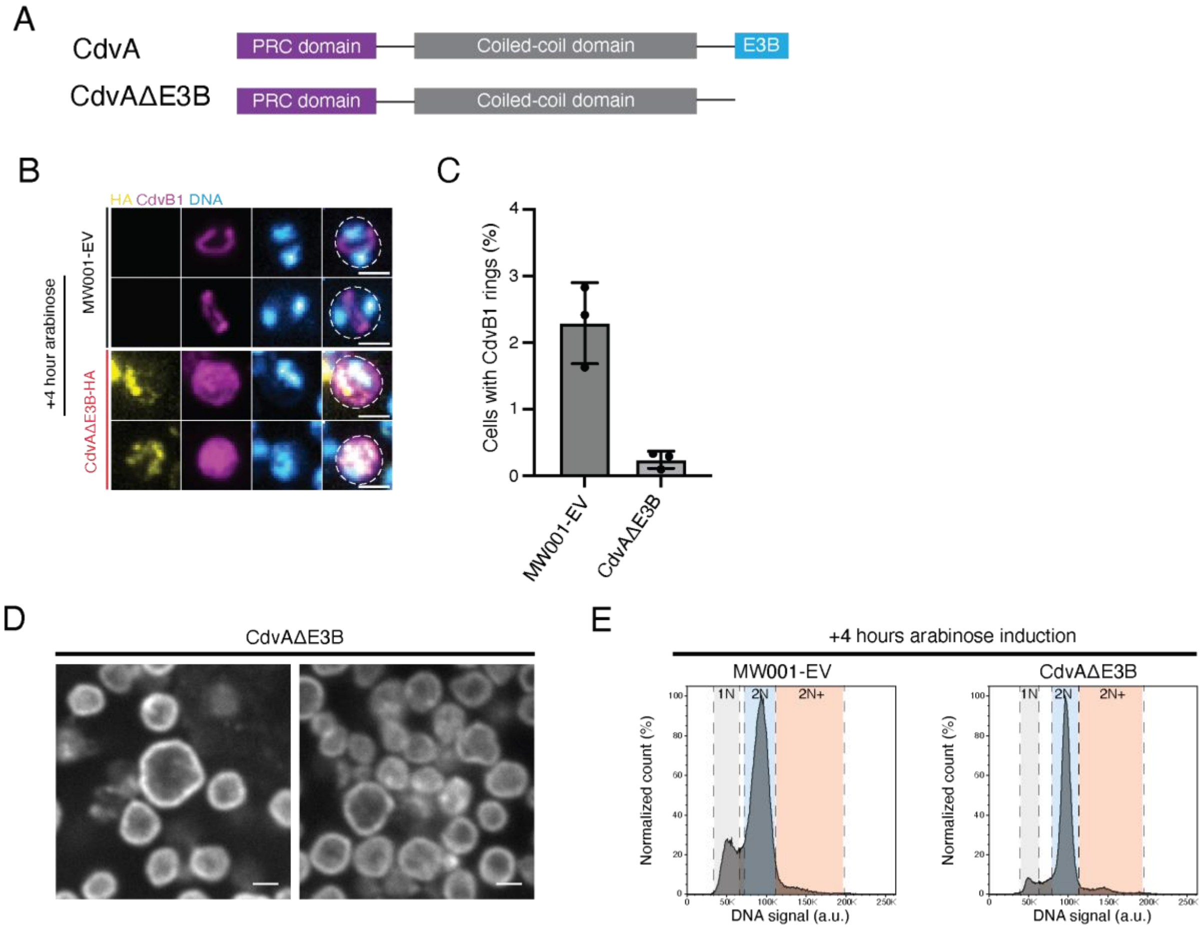

Figure S5: CdvAΔE3B blocks ESCRT-III ring formation and cell division

(A) Schematic of the full length CdvA protein and the CdvA$_{\Delta E3B}$ truncation mutant. **(B)** Representative immunofluorescent images of MW001-EV and CdvA$_{\Delta E3B}$-HA cells stained with HA and CdvB1 after 4 hours of arabinose induction. Scale bar = 1µm. **(C)** Quantification of proportion of cells that have CdvB1 rings in MW001-EV and CdvA$_{\Delta E3B}$-HA after 4 hours arabinose induction (n=>2500, N=3). **(D)** Immunofluorescent images of large CdvA$_{\Delta E3B}$-HA cells stained with ConA that have failed multiple rounds of cell division. Scale bar 1µm. **(E)** Flow cytograms of MW001 cells carrying an empty vector and those carrying a plasmid encoding CdvAΔE3B-HA after 4 hours of arabinose induction, showing a clear reduction in the G1 population in cells expressing CdvA$_{\Delta E3B}$.

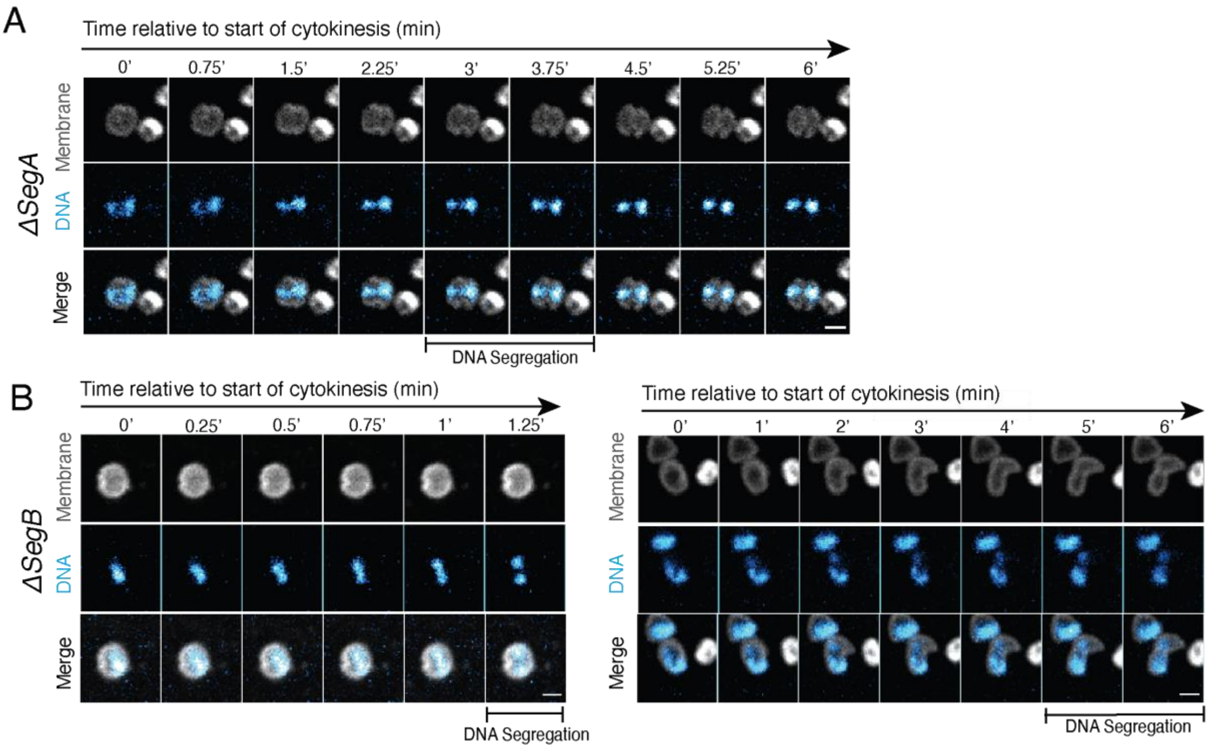

Figure S6: SegA and SegB have a role in DNA compaction

(A) Representative montage from live imaging of Δ*SegA* cells with late DNA segregation. **(B)** Representative montage from live imaging of Δ*SegB* cells with late DNA segregation and no compaction error (left) and late DNA segregation with significant DNA compaction error (right). Scale bars = 1µm.

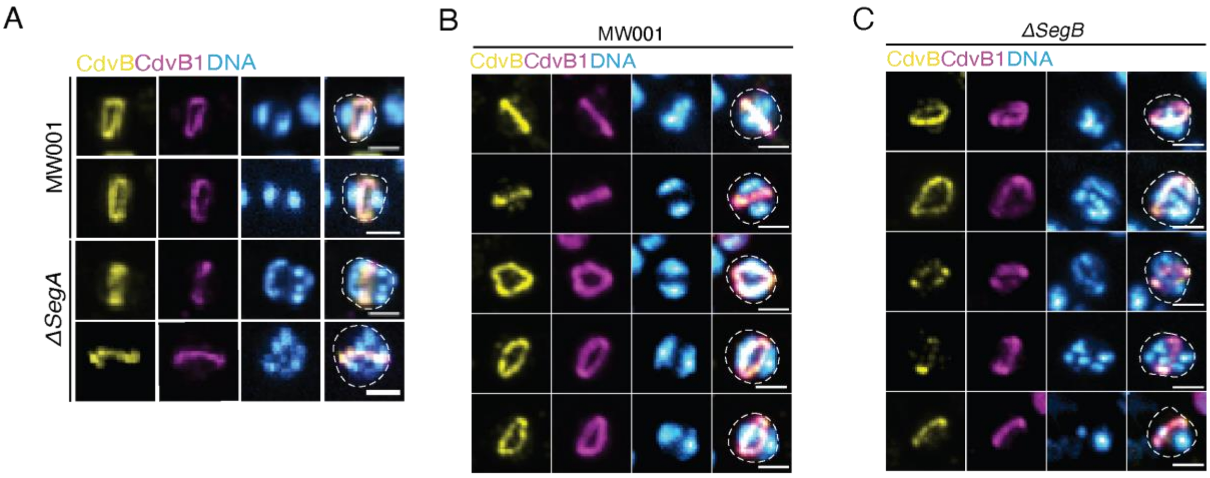

Figure S7: SegA and SegB facilitate DNA compaction at division

(A) Representative immunofluorescence images of MW001 and Δ*SegA* cells with CdvB+CdvB1 rings. **(B)** Representative immunofluorescence images of MW001 cells with CdvB+CdvB1 rings. **(C)** Representative immunofluorescence images of Δ*SegB* cells with CdvB+CdvB1 rings. Scale bars = 1µm.

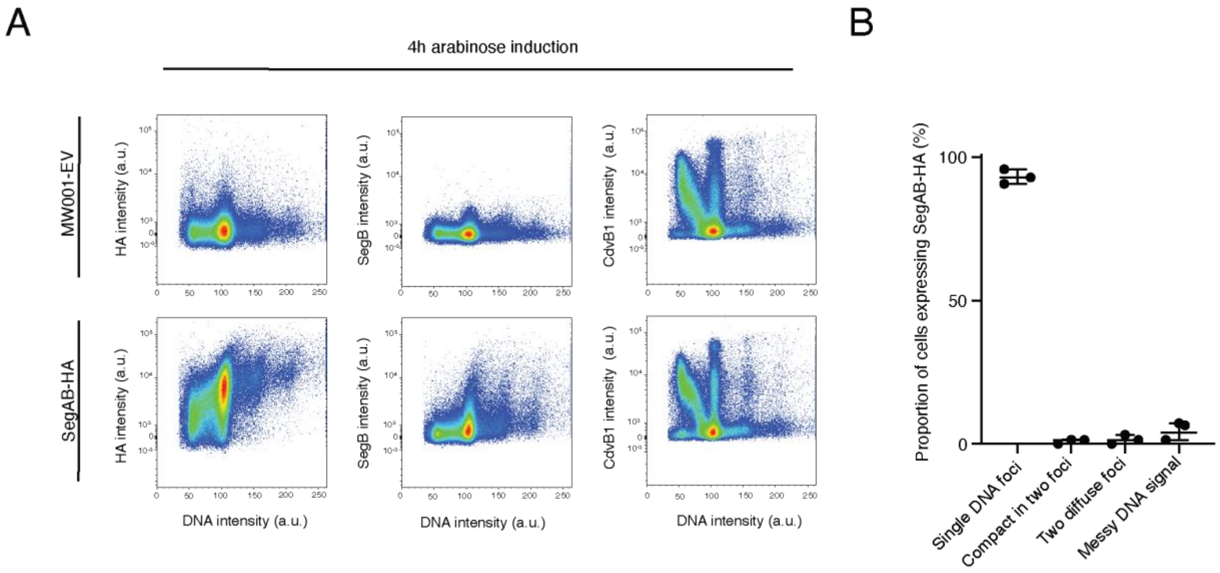

Figure S8: SegAB overexpression isn't sufficient to induce DNA segregation

(A) Flow cytograms showing the HA signal in MW001-empty vector and SegAB-HA expressing cells after 4 hours of treatment with arabinose. **(B)** Quantification of DNA segregation in cells that lack CdvB1 but which express SegAB-HA (n=337, N=3).

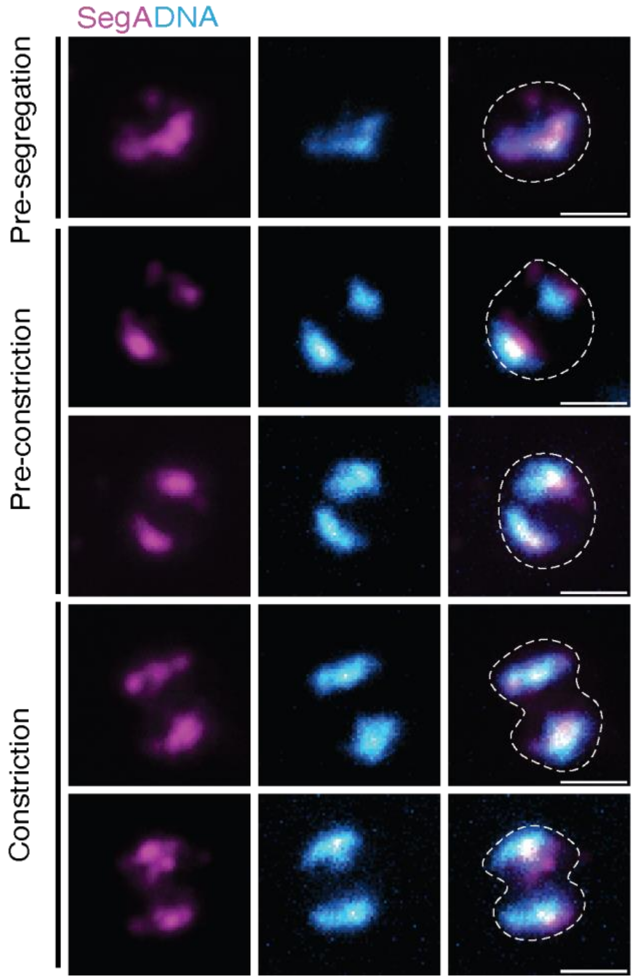

SF9: SegA staining of wildtype cells

Representative immunofluorescence images of wildtype (DSM639) cells fixed with formaldehyde and permeabilized with 0.01% SDS. Then stained with anti-SegA antibody and DAPI to visualise DNA in different stages of cell division.

Supplementary movie legends

SM1-2: Live cell imaging examples of wildtype cells (DSM639) undergoing DNA segregation and cell division (Cellmask membrane stain in grey and DNA in cyan).

SM3-5 : Live cell imaging examples of CdvAΔE3B mutants arrested in DNA mobile phase with no progression to DNA segregation or cell division (Cellmask membrane stain in grey and DNA in cyan).

Supplementary tables

Table S1: List of primary antibodies

Antibody (animal)	Supplier
Anti-CdvA (rabbit)	Lab generated
Anti-CdvA (chicken)	Lab generated
Anti-CdvB (rabbit)	Lab generated
Anti-CdvB (rat)	Lab generated
Anti-CdvB1 (chicken)	Lab generated
Anti-CdvB2 (guinea pig)	Lab generated
Anti-SegA (guinea pig)	Dyche Mullins lab
Anti-SegB (rabbit)	Dyche Mullins lab
Anti-HA (mouse)	ThermoFisher, 26183

Table S2: List of secondary antibodies

ThermoFisher	Anti-Rabbit	Anti-Chicken	Anti-Guinea pig	Anti-Mouse	Anti-Rat
Alexa Fluor 488	A11034	A11039	A11073	A10631	A11006
Alexa Fluor 546	A11035	A11040	A11074	A11030	A11081
Alexa Fluor 647	A21245	A21449	A21450	A21235	A21287

Detailed description of the computational model

Modelling interaction potentials

The two chromosome copies were modelled as bead-spring ring polymers of N_{chr} = 500 beads each. Since the genome of *Sulfolobus* comprises ≈ 3 Mbp (1), each bead represents a chromosome section of size ≈ 6 Kbp. These values are in the same range as those commonly used in coarse-grained models of bacterial chromosomes (2–7). Polymer segments are allowed to cross with a small energy penalty to simulate the action of topoisomerase-II-like enzymes (8–11). Additionally, the two chromosomes are enclosed in a thin fluid vesicle modelling the plasma membrane (12). The model used for the vesicle beads follows Yuan *et al* (12), which consists of a one-bead- thick, solvent-free fluid membrane, we use $r_{\text{min}}= 2^{1/6}\sigma$, $r_c= 2.6\sigma$, $\epsilon= 4.3kBT$, $\zeta= 4$, $\mu= 3$, $\theta= 0$, $\beta= 0$. This choice of parameters ensures that the membrane is fluid. Here, σ is the diameter of a membrane bead, which we take as the unit of length. Throughout this work, we additionally take as units of energy and mass respectively k_BT (with k_B Boltzmann's constant and T the absolute temperature) and m, the mass of a membrane bead.

Each pair of polymer beads interact with a potential

$$U_{pp}(r)= U_{pp}^{\text{hard core}}(r) + U_{pp}^{\text{soft}}(r) + U_{pp}^{\text{attr}}(r)\ (1)$$

where: $U_{pp}^{\text{hard core}}(r)$ is a hard-core repulsion term (13) introduced to prevent a complete collapse of the polymer chain in the presence of attractive interactions; $U_{pp}^{\text{soft}}(r)$ is a soft repulsive potential, which allows chain crossing (8–11); and $U_{pp}^{\text{attr}}(r)$ is a short-ranged attractive interaction (14). Here r is the interparticle distance. Similarly, polymer and membrane beads interact with the potential

$$U_{pm}(r)= U_{pm}^{\text{hard core}}(r) + U_{pm}^{\text{attr}}(r) \qquad (2)$$

These potentials have the following general expressions for two pair of beads *i, j*, where *i, j*= *p,m* for polymer and membrane beads, respectively:

$$U_{ij}^{\text{hard core}}(r) = \begin{cases} \epsilon_{ij}^{\text{hc}} \left[(\sigma_{ij}/r)^{12} - 2(\sigma_{ij}/r)^6 + 1 \right] & r \leq \sigma_{ij} \\ 0 & \text{otherwise,} \end{cases}$$

$$U_{ij}^{\text{soft}}(r) = \frac{1}{2}(E_{ij}^{\text{soft}} + \epsilon_{ij}^{\text{attr}}) \left[1 + \cos(\pi r/\sigma_{ij}) \right] \quad r \leq \sigma_{ij},$$

$$U_{ij}^{\text{attr}}(r) = \begin{cases} -\epsilon_{ij}^{\text{attr}} & r \leq \sigma_{ij} \\ -\epsilon_{\text{attr}} \cos^2 \left[\pi(r - \sigma_{ij})/2w_{ij} \right], & \sigma_{ij} \leq r < \sigma_{ij} + w_{ij} \\ 0, & \text{otherwise.} \end{cases}$$

For the polymer-polymer (*pp*) interactions, the parameter values are σ_{pp}= 2σ, ϵ_{pp}^{hc}= 4k_BT, E_{pp}^{soft}= 5k_BT, and w_{pp}= σ (width of attractive potential well). The parameter ϵ_{pp}^{attr} tunes the polymer-polymer attraction, and thus the degree of compaction of each chromosome. We note that the attraction is between any pair of beads, irrespective of which chromosome they belong to. The mass of a polymer bead is m_p= 1.1m, slightly larger than the mass of a membrane bead.

For the polymer-membrane (*pm*) interactions, the parameter values are σ_{pm}= (σ_{pp} + σ)/2= 1.5σ, ϵ_{pm}^{hc}= 4k_BT, and w_{pm}= 0.5σ. The parameter ϵ_{pm}^{attr} tunes the attraction between polymer and membrane beads, resulting in association of the polymers to the membrane; in what follows, we set ϵ_{pm}^{attr}= 0.5k_BT. The polymer-polymer and polymer-membrane potentials are shown in (Fig S3A) for the values ϵ_{pp}^{attr}= 0.25k_BT and ϵ_{pm}^{attr}= 0.5k_BT. Bonded neighbours in the same polymer chain additionally interact with an harmonic potential, $U_{bond}(r)$= $K(r - r_0)^2$, where K= 100$k_BT\sigma^{-2}$ is the bond strength and r_0= 0.7σ_{pp}= 1.4σ its equilibrium length.

Simulations

The initial state of the simulation is obtained by taking the two ring polymers (chromosomes), with each bead in a ring connected to the corresponding one in the other ring by a harmonic bond (so that the two polymers are in a “ladder-like configuration), and compressing them into a sphere until they reach volume fraction 40%. Then, the bonds between the two different rings are removed, while the intra-ring bonds are left untouched. This protocol ensures that there is initially no topological linking between the two rings. However, if linking was initially present, it would quickly be lost, since we allow for (intra- and inter-) chain crossing. We generate n_{repeat}= 50 initial configurations, which result in 50 simulation repeats for every set of parameters. The two ring polymers are then enclosed in a spherical vesicle. For most of this work, the number of vesicle beads is N_{mem}= 6750, resulting in a mean radius R= 21.4σ and a chromosome volume fraction $\phi_p \approx$ 10% inside the vesicle (Fig S3A). However, we also consider vesicles of N_{mem}= 10000 beads (R= 26.0σ, $\phi_p \approx$ 5%) and N_{mem}= 4250 beads (R= 17.1σ, $\phi_p \approx$ 20%).

Following the creation of the initial configuration, each simulation was broken up into two discrete phases, P1 and P2: P1 corresponds to the experimentally observed "mobile phase", while P2 corresponds to chromosome compaction. Below, we briefly describe these phases.

P1 (mobile phase): During this phase, the system is simulated with no polymer-polymer attraction ($\epsilon_{pp}^{\mathrm{attr}}$= 0) and no polymer-membrane association ($\epsilon_{pm}^{\mathrm{attr}}$= 0). We let the system evolve for a time T_1= $10^6\tau$, with τ= $(m\sigma^2/k_BT)^{1/2}$ the simulation unit of time, unless otherwise specified. Since during P1 there are no attractive interactions between the chromosomes, the only driving force for segregation is the entropic penalty resulting from the overlap of polymeric coils (2–4),(15–17) As discussed in the main text, this force is sufficient to lead to an appreciable degree of segregation.

P2 (compaction): During P2 we introduce both compaction and membrane association by setting the polymer-polymer attraction to $\epsilon_{pp}^{\mathrm{attr}}$= 0.25$k_BT$ and the polymer-membrane one to $\epsilon_{pm}^{\mathrm{attr}}$= 0.5$k_BT$.

The total duration of P2 is 30% that of P1, *i.e.*, T_2= $3\times10^5\tau$. Whereas membrane

association is introduced gradually in all cases, by increasing ϵ_{pm}^{attr} over $T_2/2$ time steps, we consider two protocols to introduce compaction. These are fast compaction, in which ϵ_{pp}^{attr} increases suddenly from 0 to the final value over a very short time $T_2/30 = 10^4$; and slow compaction, in which it increases gradually over the same timescale as the one chosen for membrane association, *i.e.* $T_2/2$. Since we observe experimentally that compaction happens away from the mid-cell, we only allow compaction outside of the region $L_x/2 - W/2 < x < L_x/2 + W/2$, with L_x the length of the simulation box in the x direction. Unless otherwise stated, the x direction in P2 is chosen to match with the direction of the maximum segregation axis, derived from Linear Discriminant Analysis applied to the final configuration of P1 (see *Quantification of segregation* below). Thus, the region in which compaction is inhibited lays perpendicular to the segregation axis. The parameter W represents the width of the “no-compaction” region in which compaction is inhibited: in practice, for all polymer beads in this region ϵ_{pp}^{attr} is set to zero. In this work, we take $W = 0.75R$. We also test $W = 0.5R$, finding that our results remain largely unaffected for this lower value. For $W = 0$ (uniform compaction), instead, the amount of segregation is significantly lower.

The simulations are performed at constant number of particles, volume and temperature. The solvent is treated implicitly using a Langevin thermostat(18), which also ensures that the temperature T is kept constant. The viscous friction that each bead experiences is $\zeta = 10m/\tau$. The simulations are carried out using LAMMPS(19), where time integration is performed using the velocity Verlet algorithm, with time step $\delta t = 10^{-2}\tau$. Periodic boundary conditions are applied in all three spatial directions, however this is irrelevant except for a few membrane particles very rarely being released from the vesicle. Simulations snapshots are visualized using OVITO(20).

Quantification of segregation

To quantify the amount of segregation of the two chromosome copies, we employ two different methods. In the first and most straightforward case, the distance between the centres of mass of the two chromosomes is measured (which we henceforth call DNA-DNA distance for simplicity). We normalize this quantity by dividing it by the mean vesicle radius R; this also facilitates the comparison between vesicles of different

sizes. The second method we employ is Linear Discriminant Analysis (LDA)(21), which we implement using the Python module scikit-learn(22). In practice, this amounts to trying to find a 2D plane in 3D space such that all the coordinates of the first chromosome lay to one side of the plane,with all the coordinates of the second chromosome laying to the other side. The normal vector to this plane defines what we call the *segregation axis*. If the two chromosomes are perfectly segregated, finding a plane that exactly separates their coordinates will be possible. In general, however, this procedure will result in a certain fraction f of the polymer coordinates being "misclassified", *i.e.*, laying on the wrong side of the best-fitting plane. Since for perfectly mixed coordinates $f = 1/2$ (50% of the coordinates are misclassified), we define a segregation efficiency $s = 1 - 2f$, which is equal to 0 for perfect mixing ($f = 1/2$) and to 1 for perfect segregation ($f = 0$).

Effect of chromosome volume fraction on segregation

In Fig 1E we showed the extent of segregation during the mobile phase, quantified both by the segregation efficiency and the DNA-DNA distance between the two chromosomes (see *Quantification of segregation*). The chromosome volume fraction considered in the main text is $\phi_p \approx 10\%$, which is the one that best matches the one estimated from visual inspection of the experimental images. However, since it is difficult to give a precise estimate of the volume fraction of the chromosome of *Sulfolobus*, we check here that our results are robust with respect to variations in the chromosome volume fraction. We show the results of simulations performed for $\phi_p \approx 5\%$ (Fig S3B) and $\phi_p \approx 20\%$ (Fig S3C). We observe that the extent of segregation reached at the end of the mobile phase increases slightly when the volume fraction decreases; however, the overall phenomenology remains the same. We thus conclude that our results are robust with respect to moderate changes in the chromosome volume fraction.

Effect of mobile phase duration

In the main text, we set the duration of the mobile phase to be long enough that the system reaches equilibrium, and that the extent of segregation (as measured by the segregation efficiency and the DNA-DNA distance) reaches on average a plateau. We also investigated the impact of shortening the duration of the mobile phase to 10% of its normal duration ($10^5\tau$ instead of $10^6\tau$). The results are shown (Fig S3D) where we compare the segregation efficiency and the DNA-DNA distance during fast compaction for the two cases. As expected, the initial extent of segregation is significantly lower after a shorter mobile phase. Following both a short and a regular mobile phase, compaction induces a similar increase in segregation; however, due to the lower initial value, the segregation reached after a short mobile phase is lower than the one reached after a regular mobile phase. In the snapshots (Fig S3D), we show the initial and final states for systems undergoing compaction after a regular and short mobile phase.

Effect of segregation axis alignment

As detailed in *Simulation Protocol – P2 (Compaction)*, we allow compaction only outside of a region of width W=0.75 R centered at the mid-cell. In simulations, we align this region perpendicularly to the axis of maximum segregation, which is determined using LDA. Here, we also investigated the behavior of the system during the compaction phase when the alignment of the "no-compaction" zone is instead chosen randomly. The two cases (aligned and misaligned) are compared (Fig S3E). We find that, in the misaligned case, compaction leads to an increase of segregation, but to a smaller extent than the one obtained in the aligned case. We thus conclude that compaction must take place along the axis of maximum segregation in order to reach the best possible chromosome individualization. In the snapshots in (Fig S3E), we show the initial and final states for systems undergoing fast compaction for the aligned and misaligned case.

Effect of parameter *W* (width of "no-compaction" zone)

The parameter *W* represents the width of the mid-cell region in which compaction is inhibited (see *Simulation protocol*). Throughout this work, we have set $W = 0.75R$, with R the mean vesicle radius. In (Fig S3F), we show the extent of segregation during the compaction phase, quantified both by the segregation efficiency and the DNA-DNA distance between the two chromosomes, for $W = 0.75R$, $0.5R$, and 0 (the latter corresponding to uniform compaction throughout the cell). One can see that the results remain largely unchanged for $W = 0.5R$, whereas for $W = 0$ the DNA-DNA distance is significantly lower, signaling a lower degree of segregation. In the snapshots in (Fig S3F), we show the initial, intermediate and final states of systems undergoing fast compaction with $W = 0.75R$ and $W = 0$. One can see that if $W = 0$, compaction happens uniformly throughout the cells and the two chromosomes compact into a single mass, which eventually associates to the membrane.

In *cis* compaction

In our simulations, compaction is inhibited in the mid-cell, but it involves all chromosome beads, without distinguishing between the two chromosomes. In this sense, compaction takes place both "in-*cis*" and "in-*trans*". It is well known, however, that in-cis compaction mechanisms, such as loop-extruder-mediated compaction, are required in most bacteria and eukaryotes to successfully segregate the genome(7, 10). Despite it being currently unknown whether similar mechanisms contribute to chromosome segregation in *Sulfolobus*, here we decided to study the effect of allowing only in-*cis* compaction. Instead of inhibiting compaction in the mid-cell, we explore an in-*cis* compaction mechanism where the signal for compaction propagates along the chromosome. This mechanism is chosen to mimic the action of a protein that binds to DNA and drives in-cis compaction via local spreading. However, the model is agnostic with regards to the nature of compaction mechanism at the microscopic scale.

To model in-*cis* compaction, we select the two beads, belonging each to a different chromosome, that are the furthest apart from each other in the direction of the axis of maximum segregation. Starting from the local initiator sites, we use the LAMMPS REACTER package (23) to change the interactions of bonded neighbouring beads

every N_{react} time steps, turning the interaction from purely repulsive to attractive and generating a compaction signal that propagates along the chromosome by local spreading. By changing N_{react}, we can modulate the signal propagation speed, choosing it to match the “fast” and “slow” compaction speeds of our original model. The polymer-polymer and polymer-vescicle interactions are the same used in our original model, $\epsilon_{pp}^{attr}= 0.25k_BT$ and $\epsilon_{pm}^{attr}= 0.50k_BT$.

The results of this in-cis compaction protocol are shown in Fig S3G, where we compare fast and slow in-cis compaction. In both cases, the segregation efficiency reaches ≈80%, which is significantly higher than what obtained with the original compaction protocol (≈60%, Fig 1F). Also the normalized DNA-DNA distance reaches higher values. Similarly to our original protocol, faster compaction leads to more robust segregation. However, the difference between fast and slow compaction is smaller than when in-trans compaction is allowed. These results show that in-cis compaction leads to more robust segregation, without the need to inhibit compaction in the mid-cell.

Supplemental References

1. Q. She, *et al.*, The complete genome of the crenarchaeon Sulfolobus solfataricus P2. *Proc. Natl. Acad. Sci. U. S. A.* **98**, 7835–7840 (2001).

2. S. Jun, B. Mulder, Entropy-driven spatial organization of highly confined polymers: lessons for the bacterial chromosome. *Proc. Natl. Acad. Sci. U. S. A.* **103**, 12388–12393 (2006).

3. A. Arnold, S. Jun, Time scale of entropic segregation of flexible polymers in confinement: Implications for chromosome segregation in filamentous bacteria. *Phys. Rev. E* **76**, 031901 (2007).

4. Y. Jung, *et al.*, Ring polymers as model bacterial chromosomes: confinement, chain topology, single chain statistics, and how they interact. *Soft Matter* **8**, 2095–2102 (2012).

5. J. Harju, C. P. Broedersz, Physical models of bacterial chromosomes. *Mol. Microbiol.* **123**, 143–153 (2025).

6. J. Harju, T. Armbruster, C. Broedersz, Loop-Extruder-Mediated Rigidity Can Globally Order Bacterial Chromosomes. *PRX Life* **3**, 013014 (2025).

7. J. Harju, M. C. F. van Teeseling, C. P. Broedersz, Loop-extruders alter bacterial chromosome topology to direct entropic forces for segregation. *Nat. Commun.* **15**, 4618 (2024).

8. N. Naumova, *et al.*, Organization of the mitotic chromosome. *Science* **342**, 948–953 (2013).

9. C. A. Brackley, J. Allan, D. Keszenman-Pereyra, D. Marenduzzo, Topological constraints strongly affect chromatin reconstitution in silico. *Nucleic Acids Res.* **43**, 63–73 (2015).

10. Goloborodko, A., Imakaev, M. V., Marko, J. F., Mirny, L. A. Compaction and segregation of sister chromatids via active loop extrusion. eLife 5, e14864

11. G. Forte, *et al.*, Bridging condensins mediate compaction of mitotic chromosomes. *J. Cell Biol.* **223**, e202209113 (2024).

12. Yuan, H *et al.,* One-particle-thick, solvent-free, coarse-grained model for biological and biomimetic fluid membranes. *Physical Review E* **82**, 011905 (2010)

13. Weeks, J. D., Chandler, D., Andersen, H. C. Role of repulsive forces in determining the equilibrium structure of simple liquids. The Journal of Chemical Physics, 54(12), 5237–5247 (1971).

14. I. R. Cooke, K. Kremer, M. Deserno, Tunable generic model for fluid bilayer membranes. *Phys. Rev. E* **72**, 011506 (2005).

15. Grosberg, A. Y., Khalatur, P. G., and Khokhlov, A. R. *Polymer coils with*

excluded volume in dilute solution: the invalidity of the model of impenetrable spheres and the influence of hydrodynamic interaction. *Die Makromolekulare Chemie Rapid Communications*, 3(10), 709–713. (1982)

16. Marenduzzo, D. and Orlandini, E., Topological and entropic repulsion in biopolymers. *Journal of Statistical Mechanics: Theory and Experiement.* (2009).

17. S. Jun, A. Wright, Entropy as the driver of chromosome segregation. *Nat. Rev. Microbiol.* **8**, 600–607 (2010).

18. T. Schneider, E. Stoll, Molecular-dynamics study of a three-dimensional one-component model for distortive phase transitions. *Phys. Rev. B* **17**, 1302–1322 (1978).

19. A. P. Thompson, *et al.*, LAMMPS - a flexible simulation tool for particle-based materials modeling at the atomic, meso, and continuum scales. *Comput. Phys. Commun.* **271**, 108171 (2022).

20. A. Stukowski, Visualization and analysis of atomistic simulation data with OVITO–the Open Visualization Tool. *Model. Simul. Mater. Sci. Eng.* **18**, 015012 (2009).

21. Hastie, T., Tibshirani, R., & Friedman, J. H. The Elements of Statistical Learning: Data Mining, Inference, and Prediction (2nd ed.). Springer. (2009)

22. Pedregosa et al., Scikit-learn: Machine Learning in Python. Journal of Machine Learning Research, 12, 2825–2830 (2011).

23. J. R. Gissinger, B. D. Jensen, and K. E. Wise, REACTER: A Heuristic method for reactive molecular dynamics, *Macromolecules* 53, 9953 (2020).
